# Disruption of the NlpD lipoprotein of the plague pathogen *Yersinia pestis* affects iron acquisition and the activity of the twin-arginine translocation system

**DOI:** 10.1371/journal.pntd.0007449

**Published:** 2019-06-06

**Authors:** Avital Tidhar, Yinon Levy, Ayelet Zauberman, Yaron Vagima, David Gur, Moshe Aftalion, Ofir Israeli, Theodor Chitlaru, Naomi Ariel, Yehuda Flashner, Anat Zvi, Emanuelle Mamroud

**Affiliations:** Department of Biochemistry and Molecular Genetics, Israel Institute for Biological Research, Ness-Ziona, Israel; Faculty of Science, Ain Shams University (ASU), EGYPT

## Abstract

We have previously shown that the cell morphogenesis NlpD lipoprotein is essential for virulence of the plague bacteria, *Yersinia pestis*. To elucidate the role of NlpD in *Y*. *pestis* pathogenicity, we conducted a whole-genome comparative transcriptome analysis of the wild-type *Y*. *pestis* strain and an *nlpD* mutant under conditions mimicking early stages of infection. The analysis suggested that NlpD is involved in three phenomena: (i) Envelope stability/integrity evidenced by compensatory up-regulation of the Cpx and Psp membrane stress-response systems in the mutant; (ii) iron acquisition, supported by modulation of iron metabolism genes and by limited growth in iron-deprived medium; (iii) activity of the twin-arginine (Tat) system, which translocates folded proteins across the cytoplasmic membrane. Virulence studies of *Y*. *pestis* strains mutated in individual Tat components clearly indicated that the Tat system is central in *Y*. *pestis* pathogenicity and substantiated the assumption that NlpD essentiality in iron utilization involves the activity of the Tat system. This study reveals a new role for NlpD in Tat system activity and iron assimilation suggesting a modality by which this lipoprotein is involved in *Y*. *pestis* pathogenesis.

## Author summary

We have previously shown that the NlpD lipoprotein, which is involved in the regulation of cell morphogenesis, is essential for virulence of the plague bacteria, *Yersinia pestis*. To uncover the role of NlpD in *Y*. *pestis* pathogenicity, we conducted a whole-genome comparative transcriptome analysis as well as phenotypic and virulence evaluation analyses of the *nlpD* and related mutants. The study reveals a new role for the *Y*. *pestis* NlpD lipoprotein in iron assimilation and Tat system activity.

## Introduction

Bacteria and in particular bacterial pathogens have successfully evolved sophisticated mechanisms that allow them to sense, cope and adapt to varying conditions in their immediate surroundings. The rapid detection of extracellular signals such as the concentrations of nutrients, ion sources, temperature, stress conditions and the presence of host immune cells, influence transcriptional regulatory systems that in turn lead to physiological and morphological changes that enable the organism to survive within hostile environments such as those encountered in the host during infection [1].

The Gram-negative pathogen *Yersinia pestis* is the causative agent of plague, a disease that has inflicted millions of deaths in three world pandemics [2]. Plague still persists in Africa, Asia and the Americas and as of today, it is categorized as a re-emerging disease [3]. The prevalent form of the disease is bubonic plague, which develops following transmission of the pathogen from rodent reservoirs to humans via infected fleas and has high mortality rate if untreated [4,5]. Primary pneumonic plague, which represents one of the most severe forms of the disease, is less abundant in nature and results from the inhalation of *Y*. *pestis*-containing droplets or aerosols. Pneumonic plague is a contagious rapidly progressing disease that leads to 100% mortality in untreated patients [2]. These characteristics as well as the inhalational nature of infection led to the recognition of plague as a bioterror threat agent [6].

The ability of *Y*. *pestis* to invade the mammalian host, colonize internal organs and overcome the immune response is attributed to the combined activities of multiple virulence pathways that are activated in a timely manner during infection in response to the host *milieu* signals [7,8]. Some of these pathways have been demonstrated to be absolutely required for the bacterial pathogenesis in animal models. These include molecular systems that enable the acquisition of essential nutrients during infection as well as those required for evading the host immune response such as the type 3 secretion system (T3SS) [9,10,11,12,13,14,15,16,17,18,19].

We have previously documented that the NlpD lipoprotein is essential for *Y*. *pestis* virulence in animal models of plague infection and that the *nlpD* mutant is impaired in its ability to colonize internal organs [20]. NlpD is conserved among Gram-negative bacteria and exhibits characteristic LysM and LytM domains found in enzymes involved in bacterial cell wall remodeling [21,22]. Consistent with this known biological role of LytM-containing proteins, the phenotype of the *Y*. *pestis nlpD-*disrupted mutant is characterized by altered chain-forming cell morphology [20]. Interestingly, despite its substantial virulence attenuation, the mutant was not affected in terms of *in vitro* growth or in the activity of the T3SS [20], which is essential for the pathogenicity of the bacteria. To gain further insights into the role of NlpD in the manifestation of *Y*. *pestis* virulence, we extended the characterization of the *nlpD* mutant in the present study by performing transcriptomic, phenotypic, and molecular genetic analyses. Integration of the results led to the unexpected finding that in *Y*. *pestis*, NlpD is required for iron assimilation and for the activity of the twin-arginine system (Tat) which translocates folded proteins across the bacterial cytoplasmic membrane in a wide range of bacteria [23]. Systematic deletion mutagenesis of Tat genes in the virulent *Y*. *pestis* Kim53 strain indicated that the Tat system is required for *Y*. *pestis* iron assimilation as well as virulence manifestation in the mouse plague infection models.

## Materials and methods

### Ethics statement

This study was carried out in strict accordance with the recommendation in the Guide for the Care and Use of Laboratory Animals of the National Institute of Health. All animals experiments were performed in accordance with Israeli law and were approved by the Ethics Committee for animal experiments at the Israel Institute for Biological Research (Protocols M-03-16, M-81-16, M-57-11).

### Bacterial strains, plasmids and mutant construction

The *Y*. *pestis* strains and plasmids used in this study are listed in Table 1. Construction of the Kim53 deletion mutants was performed by replacing part of the gene coding sequence with a linear fragment containing a resistance cassette by homologous recombination using previously established methodologies [16]. The sequence that was deleted from the *Y*. *pestis* genome in each mutant is indicated in Table 1.

**Table 1:** *Y*. *pestis* strains and plasmids used in this study.

*Y*. *pestis* strains and plasmid	Relevant characteristic(s)	Reference or source
**Strains**		
Kimberley53 (Kim53)	Virulent strain	[24]
EV76	Vaccine strain, Δ*pgm* strain	[15]
Kim53Δ*nlpD*	Kim53 strain in which bp 112 to 318 (out of 999) of the *nlpD* gene were deleted; kan^R^	[20]
Kim53Δ*tatA*	Kim53 strain in which bp 10 to 255 (out of 267) of the *tatA* gene were deleted; kan^R^	This study
Kim53Δ*tatB*	Kim53 strain in which bp 13 to 645 (out of 663) of the *tatB* gene were deleted; kan^R^	This study
Kim53Δ*tatC*	Kim53 strain in which bp 18 to 771 (out of 774) of the *tatC* gene were deleted; kan^R^	This study
Kim53Δ*amiC*	Kim53 strain in which bp 57 to 1198 (out of 1251) of the *amiC* gene were deleted; kan^R^	This study
Kim53Δ*nlpD*+*pnlpD*	Kim53Δ*nlpD + pnlpD* complemented strain	[20]
Kim53pTorA_signal_-GFP	Kim53 + pTorA_signal_ -GFP plasmid	This study
Kim53Δ*nlpD*pTorA_signal_-GFP	Kim53Δ*nlpD* + pTorA_signal_ -GFP plasmid	This study
Kim53Δ*tatA*pTorA_signal_-GFP	Kim53Δ*tatA* + pTorA_signal_ -GFP plasmid	This study
Kim53Δ*tatB*pTorA_signal_-GFP	Kim53Δ*tatB* + pTorA_signal_ -GFP plasmid	This study
Kim53Δ*tatC*pTorA_signal_-GFP	Kim53Δ*tatC* + pTorA_signal_ -GFP plasmid	This study
Kim53Δ*tatA ptatA*	Kim53Δ*tatA* + p*tatA* plasmid	This study
Kim53Δ*tatC ptatC*	Kim53Δ*tatC* + p*tatC* plasmid	This study
Kim53p:*gadC*:GFP	Kim53+ (pCA24N:*gadC*+GFP) plasmid	This study
Kim53p:*btuC*:GFP	Kim53+ (pCA24N:*btuC*+GFP) plasmid	This study
Kim53p:*napG*:GFP	Kim53+ (pCA24N:*napG*+GFP) plasmid	This study
Kim53Δ*nlpD*:*gadC*:GFP	Kim53Δ*nlpD* + (pCA24N:*gadC*+GFP) plasmid	This study
Kim53Δ*nlpD*:*btuC*:GFP	Kim53Δ*nlpD* + (pCA24N:*btuC*+GFP) plasmid	This study
Kim53Δ*nlpD*:*napG*:GFP	Kim53Δ*nlpD* + (pCA24N:*napG*+GFP) plasmid	This study
Kim53Δ*nlpD*+*pnlpD*:*napG*:GFP	Kim53Δ*nlpD+pnlpD* complemented strain [20] + (pCA24N:*napG*+GFP) plasmid	This study
**Plasmids**		
p*tatA*	The complete *tatA* coding sequence inserted into the pWKS30 plasmid (HindIII, XbaI), lac promoter; ampR	This study
p*tatC*	The complete *tatC* coding sequence inserted into the pWKS30 plasmid (HindIII, XbaI), lac promoter; ampR	This study
pTorA_signal_-GFP	pBR322 with TorA signal fused to GFPuv protein	[25]
pCA24N-*gadC*	Plasmid from ASKA collection (pCA24N) + *gadC* gene fused to GFP	[26]
pCA24N-*btuC*	Plasmid from ASKA collection (pCA24N) +*btuC* gene fused to GFP	[26]
pCA24N-*napG*	Plasmid from ASKA collection (pCA24N) +*napG* gene fused to GFP	[26]

Deletion of the *Y*. *pestis nlpD* gene has been described previously [20]. Deletion of the *Y*. *pestis tatA*, *tatB* and *tatC* and *amiC* genes was performed using a linear fragment containing a kanamycin resistance gene that was amplified from pKD4 plasmid and used for homologous recombination as described in [27]. Deletion of the *tatA*, *tatB*, *tatC* and *amiC* genes from the *Y*. *pestis* genome was verified by PCR analysis. TatC expression was monitored by Western blot analysis in the *tatA* and *tatB* mutants to test for possible polar effect on TatC expression (S1 Fig). The analysis indicated that *tatC* expression was not affected by the *tatA* mutation yet it was affected in the *tatB* mutant, and therefore the *tatB* mutant was excluded from the analysis documented in this report (S1 Fig).

The pTorA_signal_-GFP [25] and pCA24N-*napG* [26] plasmids were used to test the functionality of the Tat system in *Y*. *pestis*.

For complementation of the *tatA* and *tatC* deletion mutants, each of the respective *tat* genes was cloned into the low copy plasmid pWKS30 [28]. Primers pWKS-*tatA*-For and pWKS-*tatA*-Rev for the *tatA* gene and primers pWKS-*tatC*-For and pWKS-*tatC*-Rev for *tatC* gene were used for PCR amplification from *Y*. *pestis* Kim53 DNA (S1 Table). The PCR products were digested with XbaI and HindIII, and then cloned into the pWKS30 vector to generate the pWKS-*tatA* and pWKS-*tatC* amp^r^ plasmids. The plasmids were transformed into the compatible *Y*. *pestis* deletion mutant. PCR analysis verified that all the newly constructed mutants carried the pMT1, pCD1 and pPCP1 plasmids and the chromosomal *pgm* locus.

### Bacterial growth conditions

*Y*. *pestis* strains were routinely grown on brain heart infusion agar (BHIA, BD, MD USA) for 48 h at 28°C. The *nlpD* and *tat* mutants were grown on BHIA supplemented with 50 μg/ml kanamycin (Sigma-Aldrich, Israel), and all complemented *Y*. *pestis* mutant were grown on BHIA supplemented with 100 μg/ml ampicillin.

For bacterial total RNA preparation, bacterial colonies were grown on BHIA plates for 48 h at 28°C, harvested and diluted in heart infusion broth (HIB) (BD, USA) supplemented with 0.2% xylose and 2.5 mM CaCl_2_ (Sigma-Aldrich, Israel) to an OD_660_ of 0.01 and grown over night (o.n.) at 28°C in a shaker (200 rpm). The resulting cultures were diluted in fresh broth to an OD_660_ of 0.05 and allowed to grow for 5 h at 37°C. Aliquots of ~5×10^8^ colony forming units (cfu) were collected by centrifugation and the cells were immediately frozen in liquid nitrogen and stored at -70°C until use.

To assess growth under iron limiting conditions, we followed protocols established at the Perry and Fetherston laboratory [29]. Several isolated colonies grown for 48 h at 28°C on a BHIA plate were collected for o.n. growth at 28°C in PMH2 medium [30] [31]. The next day, 0.1 OD_660_ of the o.n. cultures was inoculate into fresh PMH2 grown for 6–7 h at 37°C and then diluted to 0.1 OD_660_ with fresh PMH2 and grown o.n. at 37°C. The next morning, the cultures were diluted again to 0.1 OD_660_ with PMH2 and 10μl drops containing ~10^6^ bacilli were plated on iron-depleted, gradient plates and incubated for ~50 h at 37°C. Gradient plates were prepared using square plates (USA scientific, 5668–8102) to which was added a total of 70 ml of medium was added, 35 ml for the bottom layer (poured on a slope) and 35 ml for the top layer, which was poured 24 h before performing the growth assay. The bottom layer contained 1% agarose, 1× PMH2, 20 μM MgCl_2_ and 100 μM 2,2’-dipyridyl (DIP) as a chelator. The top layer contains 1% agarose, 1× PMH2 and 20 uM MgCl_2_ (no chelator). In this manner, a DIP gradient was established ranging between 0 and 100 mM. In addition, plates containing 1% agarose, 1× PMH2, 20 μM MgCl_2_ and 80 or 100 μM DIP were prepared [32] and 10μl drops containing ~10^6^ bacilli were plated and incubated for ~50 h at 37°C as described above. To rescue bacterial growth on plates containing 100 μM DIP, iron dextran (d8517, Sigma-Aldrich) was added to the medium at a concentration of 0.5 mg/ml.

To evaluate the functionality of the Tat system, bacteria containing pTorA_signal_-GFP or pCA24N constructs (Table 1) were grown o.n. at 28°C in HIB supplemented with 0.2% xylose and 2.5 mM CaCl_2_ (Sigma-Aldrich, Israel) containing 100 mg/ml ampicillin, and the next-day cultures were diluted to OD_600_ of 0.05 into 15 ml culture and grown at 37°C. After incubation for 5–7 h, 5 ml of each culture was centrifuged (10,000 g), and the cell pellets were washed with 5 ml of double-distilled water (DDW). The cells were centrifuged and then resuspended to an OD_660_ of 0.01 with DDW. For morphological analysis, bacterial cells were washed with DDW twice and diluted to an OD_660_ of 0.01.

### RNA isolation, labeling, hybridization and microarray analysis

Total RNA was purified using the RNeasy-Mini Kit (QIAGEN) according to the manufacturer’s instructions. Seven micrograms of total RNA was used for microarray analysis of each sample using the FairPlay III microarray labeling kit (Stratagene) according to the manufacturer’s instructions. To examine changes in expression of *Y*. *pestis* genes, a custom array was used [33]. The array contains 4196 coding regions and pseudogenes out of the 4321 predicted genes of the *Y*. *pestis* CO92 strain (Acc no.: NC_003143, NCBI). Hybridization and scanning were performed as suggested by Agilent. The slides were scanned in an Agilent DNA Microarray Scanner G2505B. Images were analyzed and data were extracted using Agilent Feature Extraction software version 9.5.1.1 (FE), with linear and lowess normalization performed for each array. A reference design with two biological replicates was applied to compare the wild-type Kim53 strain and the Δ*nlpD* mutant. Statistical analysis was performed using the Limma (Linear Models for Microarray Data) package from the Bioconductor project (http://www.bioconductor.org). The processed signal resulting from the FE was read into Limma using the "read.maimages" function. Background subtraction and lowess normalization were performed within each array. A quantile normalization between arrays was applied. Standard quality control was performed using the plot functions of Limma [34]. Differential expression was assessed using linear models for designed microarray experiments. The fold changes (FC) and standard errors were estimated by fitting a linear model for each gene and applying empirical Bayes smoothing to the standard errors [34]. The FDR (false discovery rate) was controlled using the method of Benjamini and Hochberg for multiple comparisons [35,36].

The P value is the result of a one-sample Student’s t test, which was applied to the natural log of the mean of each normalized value against the baseline value of 0. Genes with differences corresponding to *P*<0.05 in either the high or the low photomultiplicator scans and that had signal-to-control or control-to-signal ratios ≥2.0 were considered to be differentially regulated. The results were submitted to the GEO depository and are available online (http://www.ncbi.nlm.nih.gov/geo/, record GSE101490).

### Quantitative real-time PCR (qRT-PCR)

For qRT-PCR analysis, 2μg of total RNA was reverse-transcribed using the Reverse Transcription (RT) System kit (Promega) with random primers according to the manufacturer’s instructions. The cDNA was used as a template for qRT-PCR with specific primers (S1 Table) using an ABI 7500 instrument (Applied Biosystems, USA) with SYBR green PCR master mix (Applied Biosystems, USA). Relative quantification was determined using an average of 2 genes: YPO1045 (*tsf* gene) and YPO1415 (*pyrD* gene), for standardization of all qRT-PCR results using the comparative (-2^ΔΔCt^) method. Forty cycles of PCR amplification were performed in duplicate for each primer set.

### Western blot analysis

For Western blot analysis, bacterial colonies were grown on BHIA plates for 48 h at 28°C, harvested and diluted in heart infusion broth (HIB) (BD, USA) supplemented with 0.2% xylose and 2.5 mM CaCl_2_ (Sigma-Aldrich, Israel) to an OD_660_ of 0.05 and grown at 37°C in a shaker (200 rpm). Bacteria (OD_660_ = 0.1) were lysed in Laemmli Sample buffer (Bio-Rad) and protein concentrations were determined using bicinchoninic acid (BCA Protein Assay Reagent, Pierce) according to the manufacturer's instructions. Equal amounts of protein were loaded and separated by SDS-PAGE. After transfer to nitrocellulose membranes, duplicate membranes were developed with anti-peptide antibodies against NlpD, Pcm [20] and TatC (see below). Probing with the primary antibody was followed by incubation of the membranes with HRP-conjugated secondary antibody (A6154, Sigma-Aldrich) visualized using the LAS-3000 imaging system (Fuji) or by IRDye 680RD-conjugated secondary antibodies (LIC-92668071 and LIC-92668070 LI-COR) visualized using the Odyssey CLx imaging system from LI-COR. The TatC anti-peptide antibodies were raised by immunizing rabbits with maleimide-activated KLH (Pierce) conjugated to the synthetic peptides CYNLVSAPLIKQLPAGAS (amino acids 41–59 out of 258aa of TatC).

### Microscopy of bacterial cultures

For the morphological analysis, 5μl bacterial aliquots were placed in an 8-well slide (#5638–01940, Bellco Glass) to dry. Cells were fixed with cold methanol (−20°C) for 15 minutes and Gram stained according to the manufacturer’s instructions (HT90A kit, Sigma-Aldrich). Images were captured under a Nikon Eclipse E200 microscope connected to a Nikon DS-Fi-1 camera at a magnification of ×400 and ×1000.

For the Tat and Sec functionality analysis, bacterial cells were mounted on poly L-lysine-treated microscope slides with Fluoromount-G (Southern Biotechnology, Birmingham, Al) and covered with a glass coverslip. The slides were examined by phase-contrast and fluorescence (fluorescein isothiocyanate filter set) microscopy. The images shown were analyzed using Zeiss LSM 710 Confocal Microscope (Zeiss, Oberkochen, Germany). Fluorescence intensity quantification of the above-mentioned markers was performed using Zen1 software, Zeiss.

For DAPI and TatC labeling of bacterial cells, approximately 10^6^ cfu where placed in a well on a DoubleCytoslide (Thermo). The cells where dried for 30 minutes and fixed with cold methanol (-20°C) for 10 minutes. The slides were then transferred to 80% cold acetone (-20°C) for 30 seconds and allowed to dry. Blocking was performed for 15 minutes with 2% BSA (in PBS). Slides where washed with DDW 3 times and then labeled with a primary αTatC antibody for 1 hour (2% BSA, 0.05% Tween 20 suspended in PBS), washed 5 times with PBS and then labeled for 15 minutes with a secondary anti-rabbit antibody labeled with Alexa 594 succinimidyl ester.

Labeling of *Y*. *pestis* cells was performed with αF1 antibodies generated against the F1 capsular protein [37] and linked to FITC or with antibodies generated against the bacterium [38] and linked to Alexa fluor 647. After labeling, the slides where washed 5 times in DDW, labeled by DAPI staining for 2 minutes, washed two times with DDW, covered and mounted with cover glasses and allowed to dry in the dark. For fluorescence microscopy, the cells where captured using a Zeiss LSM 710 confocal microscope (Zeiss, Oberkochen, Germany).

### TorA signal-GFP export assay

The assay was preformed according to Alcock *et al*., [39]. Briefly, overnight HIB culture of *Y*. *pestis* strains bearing the pTorA_signal_-GFP plasmid were grown at 28°C. Cultures were diluted the next morning to 0.1 OD_660_ and grown for 6 hours at 37°C. Cells were harvested and washed twice in 10 mM Tris.Cl, 150 mM NaCl, pH 7.3.

Equal volumes of the cell suspensions (10 OD_660_) were then centrifuged, and the cell pellets resuspended in 1ml SET buffer (17.12% sucrose (w/v), 3 mM EDTA, 10 mM Tris.Cl, pH 7.3), and left at room temperature for 20 min. Cell were concentrated in the 2 ml Eppendorf tubes at maximum speed for 10 min (20,000x g). The cell pellet was resuspended in 250 μl lysozyme (3 mg/ml in water) and 750 μl ice-cold water and incubated for 20 min at 37°C. Spheroplasts were released from the periplasm by centrifugation at maximum speed for 10 min (20,000x g). Samples were analyzed by immunoblotting for GFP (αGFP antibodies, G1546, sigma-aldrich, Israel) or the cytoplasmic marker protein Pcm [20]. The data presented is representative of at least two independent experiments.

### Sec translocon assay

Assessment of Sec functionality in *Y*. *pestis* strains was performed following transformation with two plasmids from the ASKA collection [26] encoding the Sec substrates BtuC and GadC fused to GFP. These plasmids were a kind gift from professor Eitan Bibi [40]. *Y*. *pestis* wild-type and Δ*nlpD* strains were grown on BHI agar plates with chloramphenicol (25 mg/ml) for 48 h. The cells were resuspended to a final concentration of 0.2 OD660/ml in PBS. Fluorescence of the Δ*nlpD* was quantified using a Victor3 (PerkinElmer) instrument with wavelength of 485nm (excitation) and 535nm (emission) and presented relatively to the wild type strain [40].

### Animal studies

Female CD1 mice (5–6 weeks old) were purchased from Charles River (UK) and maintained under defined flora conditions at the animal facilities of the Israel Institute for Biological Research. The subcutaneous infections were performed as described previously [41]. Briefly, bacterial colonies grown on BHIA plates for 48 h at 28°C were harvested and suspended in saline solution to the required infection dose and quantified by counting cfu after plating and incubating on BHIA plates (48 h at 28°C). The intranasal (i.n.) infections were performed as described previously [20]. Briefly, bacterial colonies were grown in HIB supplemented with 0.2% (+) xylose and 2.5 mM CaCl_2_ and incubated overnight at 28°C. Cultures were diluted in saline solution to the required infection dose and quantified by cfu counting. Prior to infection, mice were anaesthetized with a mixture of 0.5% ketamine HCl and 0.1% xylazine was injected intraperitoneally followed by i.n infection with 35 μl/mouse of the bacterial suspension. The LD_50_ experiments were performed using groups of five mice. The LD_50_ values were calculated according to the method described by Reed and Muench [42],[15].

To evaluate the complementation of virulence by iron supplementation, mice (n = 6) received 5 mg iron-dextran (d8517, Sigma-Aldrich) intraperitoneally (i.p) 2 to 3 h before s.c. inoculation of 1×10^7^ cfu of the *Y*. *pestis* strains. Beginning on the second day post-infection, iron dextran (5 mg/mouse) was administered once daily i.p. during the course of the experiment.

### Statistical analysis

Statistical significance was measured using the Student’s unpaired t test. Survival curves were compared using the log-rank test. In all analyses, *p* values equal to 0.05 served as the limit of significance. Calculations were performed using GraphPad Prism 5 statistical pack.

## Results

### Transcriptomic analysis and functional classification of the differentially expressed genes in wild-type and Δ*nlpD Y*. *pestis* strains

To elucidate pathways by which NlpD is required during plague infection, we compared the transcriptional profiles of the parental virulent *Y*. *pestis* Kim53 strain and its isogenic *nlpD* mutant grown under conditions reminiscent of early stages of infection of the mammalian host.

Total RNA was prepared from *Y*. *pestis* cultures grown at 28°C, transferred to 37°C for five hours and then used as template for cDNA synthesis. Fluorescently labeled cDNA served as a probe for hybridization to a custom *Y*. *pestis* array [33]. A total of 220 genes were differentially expressed in the *nlpD* mutant (≥2-fold change in two experimental repeats, *P*<0.05) compared with the *Y*. *pestis* Kim53 strain. Among these genes, 113 were up-regulated (S2 and S4 Tables, S2A Fig) and 107 were down-regulated (S3 and S5 Tables, S2B Fig) in the mutant strain. The microarray data were validated by qRT-PCR analysis of the mRNA levels of selected genes. Plotting the data obtained using two complementary approaches revealed a strong positive correlation, confirming the microarray results (R^2^ = 0.846, see Supplementary S3 Fig, S6 Table).

Inspection of the functional annotation of the 220 differentially expressed genes (inferred from the NCBI and KEGG databases (https://www.ncbi.nlm.nih.gov/gene/ and http://www.genome.jp/kegg, respectively) indicated that membrane stress response was induced in the *nlpD* mutant (S2 Table) and iron-related metabolic pathways as well as transport systems for nutrients such as sulfate, arginine and sugar were differentially regulated (S2 Table and S3 Table). Two of the genes that were most significantly up-regulated in the mutant strain were *cpxP* and *pspA* (15.82-fold and 3.5-fold, respectively, S2 Table), belonging to the membrane stress response pathways Cpx (conjugative plasmid expression) and Psp (phage shock protein). These two pathways are involved in maintaining the homeostasis of the cytoplasmic membrane and preventing damage resulting from the accumulation of misfolded proteins in the periplasm [43,44]. These results suggest that in the absence of the NlpD lipoprotein, misfolded protein accumulation is increased in the periplasm. Activation of the Cpx and Psp stress response pathways may therefore represent a compensatory modality for retaining the integrity of the *nlpD* mutant membranes.

As indicated, many of the differentially modulated genes in the *nlpD* mutant were related to iron metabolism (~ 20%). Iron is an essential nutrient for most pathogenic bacteria and for *Y*. *pestis* in particular [45,46]. Some iron uptake systems involve an outer membrane receptor, a periplasmic binding protein and an inner membrane ATP-binding cassette (ABC) transporter. Coupling of the proton motive force of the cytoplasmic membrane to the outer membrane by the TonB, ExbB, and ExbD proteins provides the energy required for transport. Interestingly, genes that are up-regulated in the *nlpD* mutant (S2 Table) included the *exbBD-tonB* genes as well as the *ybtA* transcriptional regulator of the major iron acquisition system Yersiniabactin (Ybt) and the *irp5* gene of this system required for synthesis of the Yersiniabactin siderophore [47]. Genes encoding additional iron uptake and storage systems such as, Yiu, Fit and the ferrichrome and ferrisiderophore receptor proteins were also up regulated in the *nlpD* mutant. These results, which indicate a compensatory up-regulation of iron uptake systems, strongly suggest that the *nlpD* mutant has a limited ability to acquire iron from the environment. Inspection of the down-regulated genes in the *nlpD* mutant suggest that in response to the apparent shortage of iron, deletion of the *nlpD* gene results in the decreased expression of several iron-containing proteins (fumarate reductase, dimethyl sulfoxide reductase and nitrate reductase) as well as the expression of the IscR transcription factor, which has been shown to modulate the expression of iron-sulfur protein clusters in *Escherichia coli* [48]. Similarly, the expression of proteins that are active in metabolic processes involving Fe-S-containing proteins was down regulated probably to preserve the small amount of intracellular iron for more essential metabolic pathways (S3 Table). The suggested paucity of iron in the *nlpD* mutant is also supported by the observed decreased expression of the *ccmA-ccmH* gene cluster (S3 Table) encoding a heme export system that functions in *E*. *coli* in cytochrome c maturation [49]. Iron is required for the activity of many enzymes of the tricarboxylic acid cycle, the cytochromes, non-heme iron electron carriers of the electron transport chain and nitrogen assimilation [50]. Indeed, metabolic pathways that utilize enzymes that are co-factored by iron were down-regulated in the *nlpD* mutant, leading to a metabolic shift in comparison to the wild-type strain (S3 Table). Taken together, these results strongly suggest that the *nlpD* mutant has a limited ability to acquire iron from the environment, a characteristic which could not have been anticipated solely from the documented function of NlpD in cell wall remodeling.

### *Y*. *pestis* NlpD lipoprotein is required for growth under iron-limiting conditions

**Fig 1:**
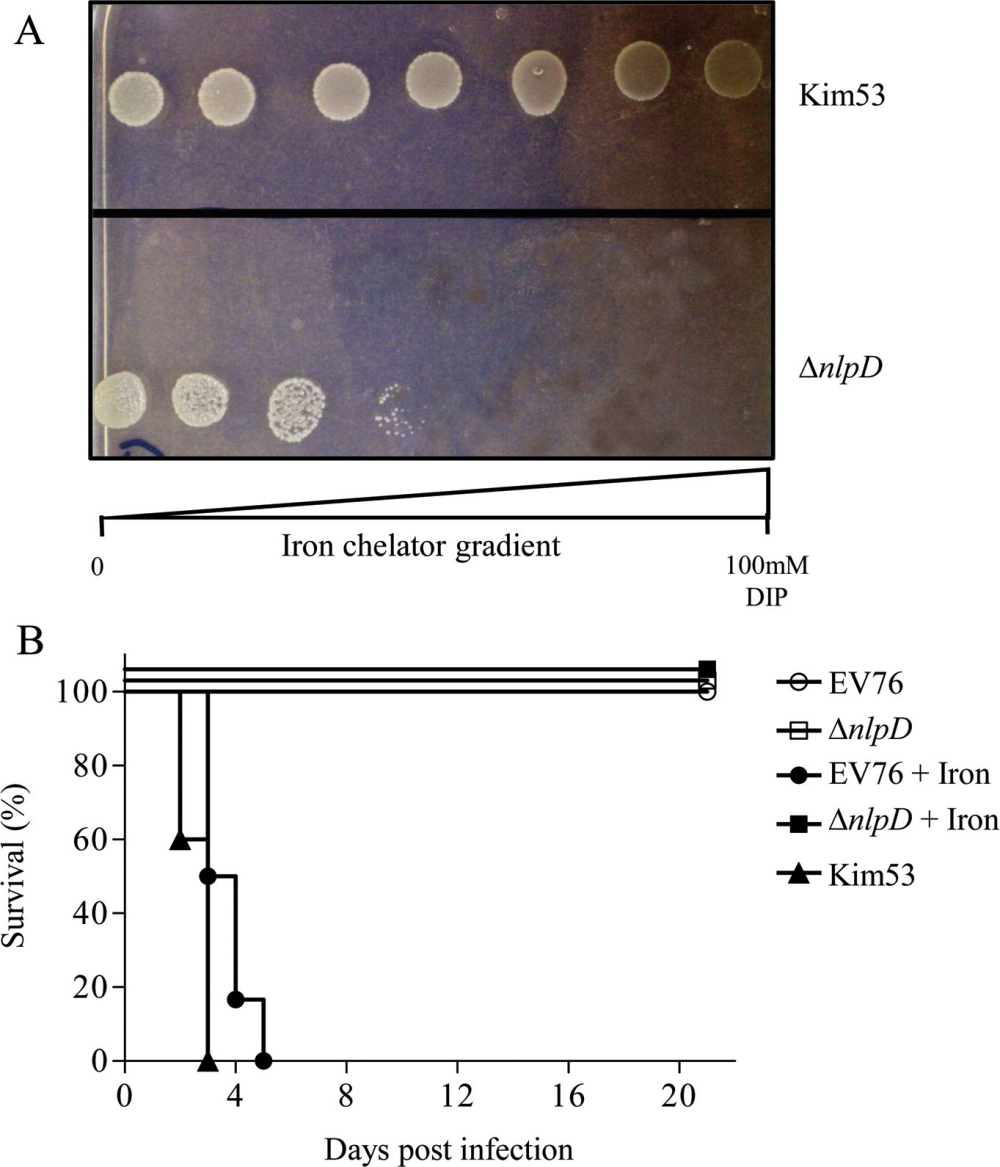
Role of iron in *nlpD* mutant growth and pathogenesis. A. Growth of *Y*. *pestis* strains under iron-limiting conditions. Kim53 (upper panel), Kim53Δ*nlpD* (lower panel). The displayed data is one representative experiment. B. Survival curves of iron-treated mice infected with attenuated *Y*. *pestis* strains. Two groups of 12 mice were infected with the attenuated *Y*. *pestis* EV76 (circles) or Kim53Δ*nlpD* (square) strains (s.c. infection with 10^7^ cfu/mouse). In each group, six mice were treated with iron dextran (filled symbols), and six mice served as control (open symbols). Survival curve of mice infected with the *Y*. *pestis* Kim53 strain (s.c. infection with 10^6^ cfu/mouse, triangles).

To confirm the hypothesis drawn from the transcriptomic analysis that the *nlpD* mutant has limited iron assimilation, the *nlpD* mutant and the parental Kim53 strains were analyzed using an *in vitro* growth assay under iron-limiting conditions [29]. Indeed, growth of the *nlpD* mutant was impaired in comparison to the wild-type strain under the iron-deficient conditions induced by addition of the iron-chelating agent 2,2’-dipyridyl (DIP) to the PMH2 defined medium (Fig 1A). Consequently, the limitation of the *nlpD* mutant to acquire iron during growth could also be manifested *in vivo* during infection and therefore may represent the cause for its avirulent phenotype in the mouse model of plague. To directly address this possibility, reversion of the avirulent phenotype of the *nlpD* mutant was attempted by exogenous administration of iron to the *Y*. *pestis nlpD*-infected mice. Such an approach has been previously described for studying the phenotype of an EV76 attenuated *Y*. *pestis* strain lacking the Ybt iron acquisition system carried in the *pgm* genomic region [51]. Thus, mice were subcutaneously infected with a single dose of 1×10^7^ cfu of EV76 or with a similar dose of the attenuated *nlpD* mutant. As shown in Fig 1B, the virulence of the EV76 strain, but not of the *nlpD* mutant, was restored by daily injection of iron dextran.

### *Y*. *pestis* NlpD lipoprotein is required for functionality of the twin-arginine translocation system

A possible explanation for the inability to revert the attenuated phenotype of the *nlpD* mutant by exogenous addition of iron is the existence of additional defects that prevent establishment of infection by the *nlpD* mutant. Apart from attenuation of virulence, the *Y*. *pestis nlpD* phenotype has thus far been characterized to include, impaired cell septation, slight sensitivity to acidic stress conditions, and the above deficiency in iron acquisition (Fig 1A) [20]. This set of phenotypes was reminiscent of the phenotypes characterizing the twin-arginine translocation system (Tat) mutants of Gram-negative bacteria [52]. The Tat system typically consists of three cytoplasmic membrane proteins: TatA, TatB, and TatC, which are encoded by a single operon and are responsible for the transport of folded proteins across the cytoplasmic membrane [53,54]. Proteins that are translocated by the Tat system include cofactor-containing enzymes, multimeric proteins that require assembly prior to export as well as integral membrane proteins [53,55,56,57]. Tat substrates function in energy metabolism, cellular division, motility and adaptation to environmental stress [52,58]. The system has been shown to be important for virulence in many bacterial pathogens including *Yersinia* species [25,52,59,60,61].

**Fig 2:**
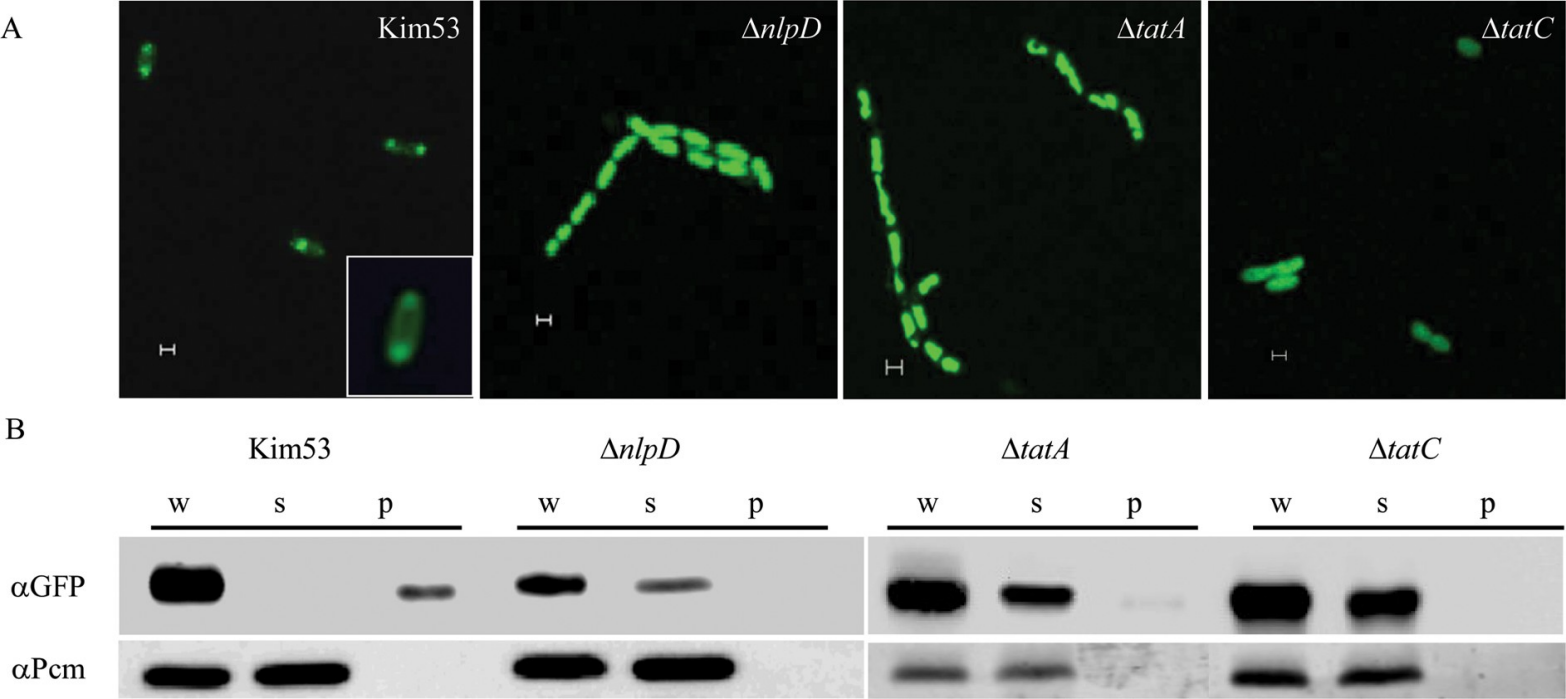
Intracellular localization of the TorA_signal_-GFP Tat-reporter protein in *Y*. *pestis* strains. A. *Y*. *pestis* strains Kim53:pTorA_signal_-GFP, Kim53Δ*nlpD*:pTorA_signal_-GFP Kim53Δ*tatA*:pTorA_signal_-GFP, and Kim53Δ*tatC*:pTorA_signal_-GFP, were inspected under a fluorescence microscope for identification of TorA_signal_-GFP localization. The scale bar represents 1 μm. B. *Y*. *pestis* cells overproducing TorA_signal_-GFP were fractionated into Whole cell (W), spheroplast (S) and periplasm (P), and were subject to immunoblotting with antibodies against GFP or against the cytoplasmic protein Pcm [20]. The blots were derived from the same experiment and were processed in parallel. The data presented are representative of at least two independent experiments and the displayed data is one representative experiment.

To examine the functionality of the Tat system in the *nlpD* mutant, an established method based on the GFP reporter protein fused to the TorA Tat signal was used [62]. In the wild type *Y*. *pestis* background, GFP was localized to the periplasm and enriched at the cell poles whereas in *Y*. *pestis* TatA and TatC mutants, localization to the periplasm and poles was lost and the GFP reporter protein was diffused completely throughout the cytoplasm (Fig 2A). This observation is consistent with previous observations for other bacterial pathogens [25,60]. In the *nlpD* mutant, the GFP reporter was completely diffused throughout the cytoplasm, as observed for the Tat mutants, suggesting loss of Tat activity (Fig 2A). Of note, mutation of the *tatB* gene resulted in a polar phenotype abrogating expression of both TatB and TatC subunits (see Materials and Methods), therefore the *tatB* mutant was excluded from the current analysis. To further confirm the loss of Tat functionality in the *nlpD* mutant strain, the cellular localization of NapG, an additional Tat-substrate protein was investigated [60]. Accordingly, a hybrid protein consisting of the full length NapG protein and a C-terminal fused GFP reporter [26], was expressed in the parental wild-type, Δ*nlpD* mutant, as well as in the NlpD trans-complemented strain. The data (S4 Fig) clearly establish that the *nlpD* mutation is accompanied by the loss of fluorescence polarity that is characteristic for the Tat dysfunctionality. Furthermore this disturbed localization was fully alleviated upon trans-complementation of the *nlpD* mutant with an NlpD-expressing construct (S4 Fig) that was also shown to restore the wild-type cell morphology and virulence phenotype [20].

The direct assessment of Tat-substrate localization in *nlpD* mutant cells by microscopy described above was further substantiated by inspection of subcellular localization of a Tat substrate reporter in Tat mutant strains which were engineered by specifically disrupting expression of each of the Tat subunits (see Table 1). Thus, the functionality of the Tat system was interrogated implementing the molecular approach [39,63] consisting of Western blot analysis of fractionated material obtained from the various mutants as well as the parental strain. The Western-blot analysis of the subcellular fractions of the *Y*. *pestis* strains clearly confirmed that Tat transport was substantially affected in the *nlpD* mutant as seen for the *tatA* and *tatC* mutants (Fig 2B, S5 Fig). These results indicated, as anticipated, that the Tat system is not functional in the *nlpD* mutant and that the *Y*. *pestis* NlpD lipoprotein is required, directly or indirectly, for Tat system functionality.

**Fig 3:**
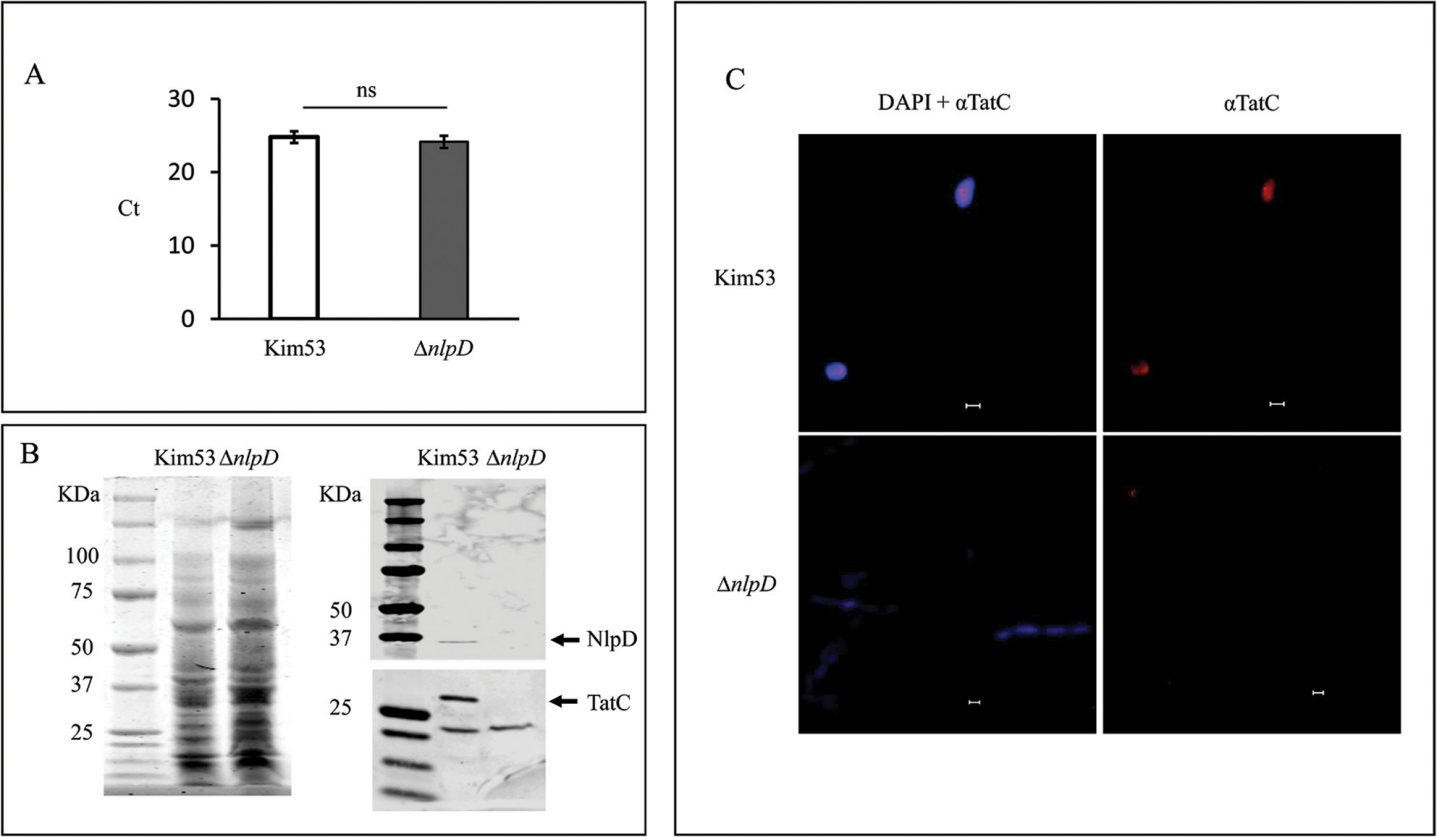
Expression of TatC in the *Y*. *pestis* strains. A. Quantitative RT-PCR analysis of *tatC* mRNA levels. mRNA from Kim53 (white histogram) and the *nlpD* mutant (gray histogram) was subjected to qRT-PCR analysis of *tatC* gene expression. The relative mRNA level was determined by calculating the threshold cycle (ΔCt) of target genes via the classic ΔCt method [90]. The results presented are an average of three independent experiments. B. Western blot analysis of TatC protein levels in total cell lysates of the wild-type Kim53 strain and the *nlpD* mutant. Whole cell lysates (10^6^ cfu/lane grown at 37°C), were subjected to Western blot analysis using anti-NlpD and anti-TatC antibodies. The Coomassie blue stained gel and the blots were derived from the same experiment and were processed in parallel. C. Distribution of TatC protein on the bacterial membrane of *Y*. *pestis* strains. Fluorescence microscopy images of wild-type Kim53 (top panel) and the *nlpD* mutant (lower panel) are presented after TatC staining alone (right panel) or with DAPI staining (left panel). Images (100×) were captured with a Zeiss LSM 710 confocal microscope (Zeiss, Oberkochen, Germany). Scale bar = 1 μm. The inset shows a magnification (×3) of stained bacterial cells. The data presented are representative of at least two independent experiments and the displayed data is one representative experiment.

Interestingly, the mRNA levels of all Tat genes (TatA, TatB and TatC) quantified in the *nlpD* mutant were indistinguishable to those of the wild-type strain, according to the microarray transcriptome analysis (http://www.ncbi.nlm.nih.gov/geo/, record GSE101490) and unambiguously confirmed by the qRT-PCR quantification of *tatC* mRNA (Fig 3A). However, Western blot analysis of TatC protein (used as a representative component for the Tat system) indicated that the protein level was reduced in the *nlpD* mutant in comparison to the wild-type strain (Fig 3B, S6 Fig). A dramatic difference between the wild type strain and the *nlpD* mutant was also observed in the confocal microscopy images of bacterial cells labeled with anti-TatC antibodies (Fig 3C). In these images, regions of fluorescence identified by anti-TatC antibodies were clearly visible throughout the cytoplasmic membrane of DAPI-stained wild-type *Y*. *pestis* bacteria (Fig 3C, upper panel), whereas no signal could be detected in DAPI-stained cells of the *nlpD* mutant (Fig 3C, lower panel).

Overall, these observations suggest that whereas the transcript levels of *tat* genes were not altered due to NlpD deletion, the *nlpD* mutant strain exhibited decreased levels of the Tat protein as indicated by monitoring TatC. These data raise the possibility that inactivation of the Tat system in the *nlpD* mutant could result from post-transcriptional events that affect proteins of the Tat machinery.

**Fig 4:**
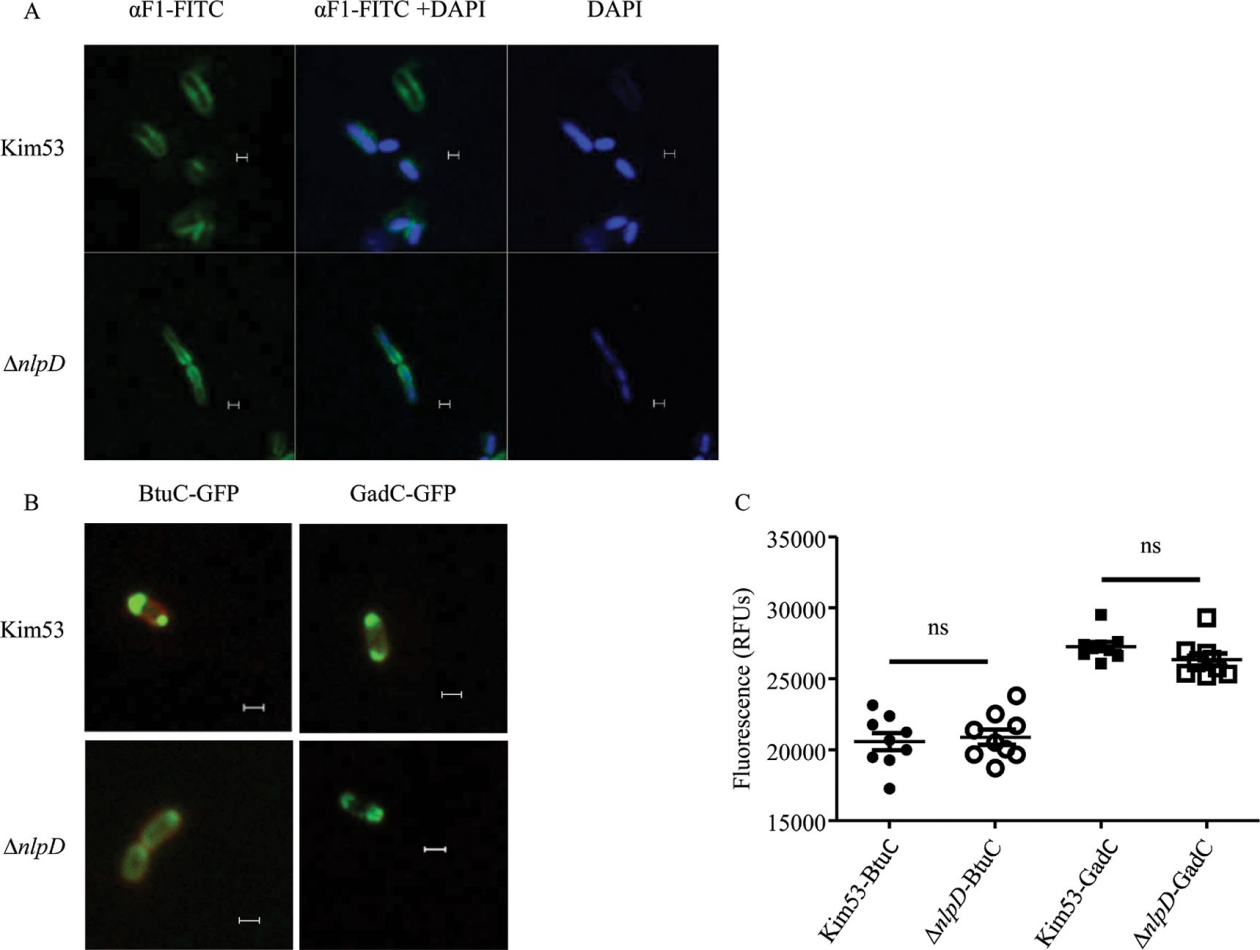
The Sec translocon is operational in the Δ*nlpD* mutant. A. The indicated bacterial cells were labeled with FITC-conjugated α-F1 antibodies (left), DAPI (right) or both α-F1 and DAPI (middle). B. Fluorescence microscopy of *Y*. *pestis* strains expressing the Sec-substrates BtuC and GadC fused to GFP. Scale bar = 1 μm. C. Relative fluorescence units (RFUs) of the Δ*nlpD* mutant expressing BtuC-GFP or GadC-GFP, compared to the wild Kim53 strain expressing the reporter proteins, according to [40]. Ns, non-significant (Unpaired t test). The data presented are representative of at least two independent experiments and the displayed data in A and B is one representative experiment.

To verify that the loss of Tat functionality did not result from a general destabilization of the cytoplasmic membrane, the activity of Sec machinery, an additional inner-membrane imbedded transport system, was assessed. The Sec machinery is essential for the transport to the periplasm of the *Y*. *pestis* F1 capsular protein [64,65]. As depicted in Fig 4A, a similar distribution of the F1 protein was observed in the outer membrane of the wild-type strain and the *nlpD* mutant. In addition, the Sec pathway substrates BtuC and GadC were effectively expressed and transported to the periplasm of both wild-type and *nlpD* strains (Fig 4B and 4C), indicating that the Sec translocon is operational in the *nlpD* mutant in a similar manner to the wild-type strain. These results strongly suggest that the loss of Tat translocation activity in the *nlpD* mutant did not result from a general destabilization of the cytoplasmic membrane.

### The *Y*. *pestis* mutants of the Tat system exhibit virulence attenuation and restricted growth under iron limiting conditions

As mentioned above, one of the major phenotypic characteristics of the *nlpD* mutant strain is its virulence attenuation. Therefore, we addressed the possibility that the NlpD associated effect on pathogenicity maybe attributed to the dysfunctionality of the Tat system. Indeed, it has been shown that the TatA protein is important for *Y*. *pestis* virulence in mouse models of plague [61]. Thus, we further addressed the attenuation of virulence and other phenotypic characteristics associated with the deletion of Tat proteins, in mutant strains exhibiting disruption of Tat genes in comparison to the phenotype exhibited by the *nlpD* mutated strain. Accordingly, the various mutants were characterized with respect to their morphology, iron acquisition capability and virulence in mouse models of plague.

**Fig 5:**
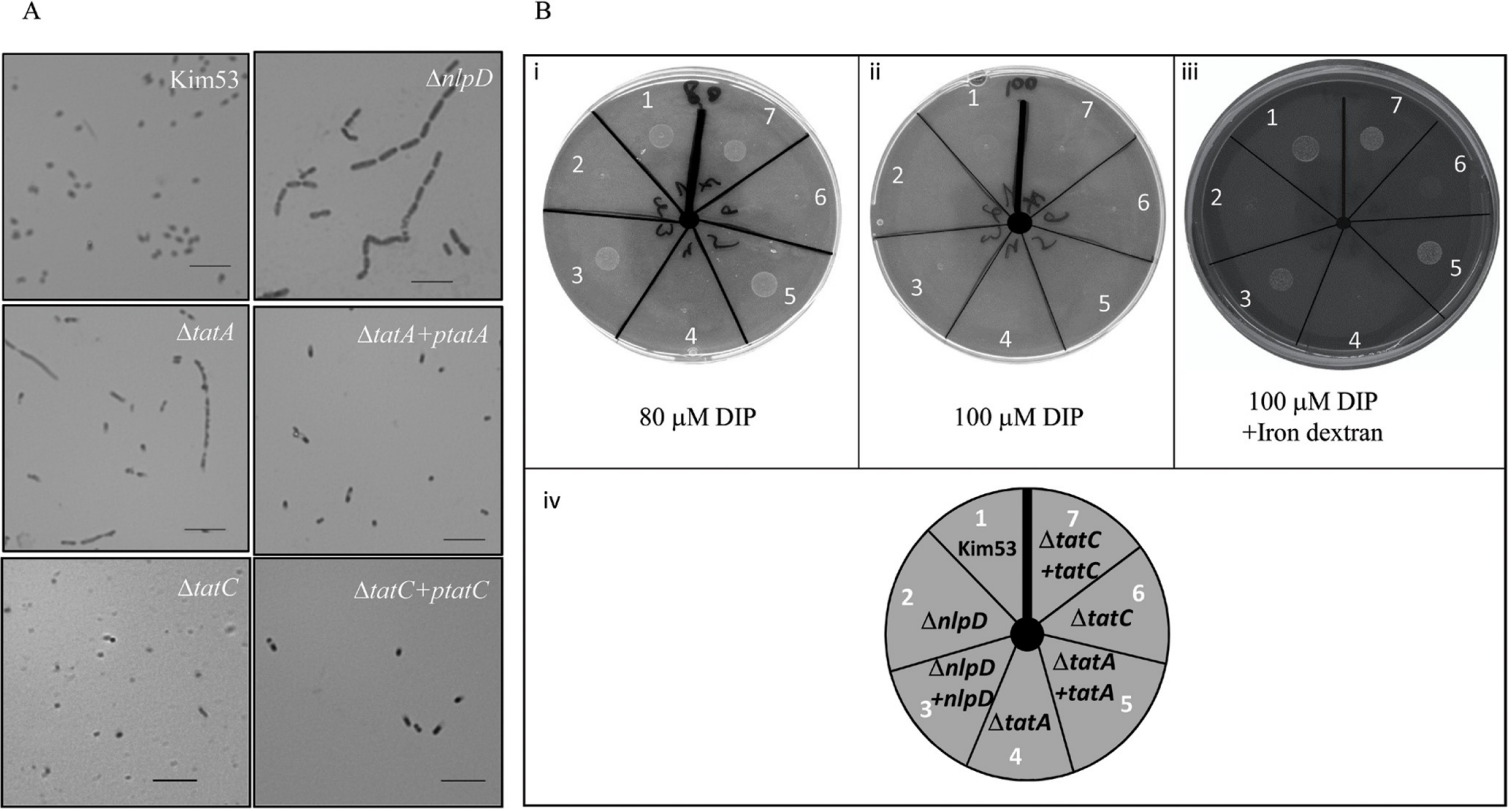
Phenotypic characterization of *Y*. *pestis tat* mutants. A. Gram staining of *Y*. *pestis* strains wild-type Kim53, Δ*nlpD*, Δ*tatA*, Δ*tatC*, Δ*tatA*+pt*atA* and, Δ*tatC*+pt*atC* was performed, and bacilli were observed by light microscopy at a magnification of ×1000. Scale bar = 10 μm. B. Growth of *Y*. *pestis* strains under iron-limiting conditions. *Y*. *pestis* strains (see description in the lower panel, iv), were grown under iron-limiting conditions (see Materials and Methods). The medium included: 1% agarose, 1× PMH2, 20 μM MgCl_2_ and 80 μM DIP (i), 100 μM DIP (ii) or 100 μM DIP with addition of iron dextran (0.5mg/ml, iii). The displayed data is one representative experiment.

Microscope analyses revealed a defect in cell segmentation of the *tatA* deletion mutant (Fig 5A). In addition, growth of the *tatA* and *tatC* mutants was severely inhibited under iron-limiting conditions in comparison to the wild-type strain, similar to the *nlpD* mutant (Fig 5Bi, Fig 1 and Table 2). Trans-complementation of the *nlpD*, *tatA* and *tatC* mutants with each of the corresponding genes (*nlpD* or *tatA* or *tatC*, respectively) was efficient in alleviating the growth under these conditions (Fig 5Bi and Table 2). Increasing the amounts of the iron chelator (DIP) to 100 μM resulted in growth inhibition of all *Y*. *pestis* strain (Fig 5Bii and Table 2). Addition of iron dextran (0.5mg/ml) to the plates containing the high concentration of DIP restored growth of the parental Kim53 strain as well as the trans-complemented *nlpD*/p*nlpD*, *tatA*/p*tatA* and *tatC*/*ptatC*, but not of *tatA*, *tatC* and *nlpD* mutants (Fig 5Biii and Table 2). Growth of the *tatA*, *tatC* and *nlpD* mutants was inhibited under iron limiting conditions and was not restored by addition of iron but only upon trans-complementation with the relevant gene (*nlpD* or *tatA* and *tatC* respectively, Fig 5Biii, Table 2).

**Table 2:** Growth of *Y*. *pestis* strains under iron-limiting conditions.

Strain	Trans-complementation	Growth^a^		
		80μg DIP	100μg DIP	100μg DIP + iron dextran
Kimberley53		+	-	++
Kim53Δ*nlpD*	-	-	-	-
Kim53Δ*nlpD*p*nlpD*	*nlpD*	+	-	++
Kim53Δ*tatA*	-	-	-	-
Kim53Δ*tatA*p*tatA*	*tatA*	+	-	++
Kim53Δ*tatC*	-	-	-	-
Kim53Δ*tatC*p*tatC*	*tatC*	+	-	++

^a^ Growth under iron limiting conditions was evaluated on the basis of an arbitrary scale; accordingly, 3 levels of growth were defined: -, +, ++.

Virulence of the *Y*. *pestis tat* mutants was evaluated in the well-established mouse models of bubonic and pneumonic plague [17,66]. As shown in Table 3, infection of CD-1 mice with high doses of the *tatA* mutant via the subcutaneous and intranasal routes was non-lethal. Furthermore, the *tatA* mutated bacteria could not be detected in the draining lymph node or the spleen of mice 48 hours after s.c. inoculation with high doses (10^7^ cfu/mouse), similar to the observations reported with respect to the *nlpD* mutated bacteria [20]. The results obtained with the s.c. administration of the TatA mutant are consistent with those of Bozue and colleagues [61], who showed that a *Y*. *pestis* CO92 *tatA* mutant was severely attenuated upon subcutaneous infection of Swiss Webster mice. As with the *nlpD* mutant (Fig 1B), the avirulent phenotype of the Kim53*tatA* mutant could not be reversed by exogenous administration of iron to the infected mice in line with the *in-vitro* growth results (Fig 5Biii). Yet, complementation by episomal expression of the *tatA* gene which restored the parental cell morphology and growth on iron-depleted medium (Fig 5 and Table 2) also reverted the virulent phenotype (Table 3), confirming that the observed phenotype is attributed to abrogation of TatA expression.

**Table 3:** Virulence of *Y*. *pestis* strains in mouse models of bubonic and pneumonic plague.

	LD_50_ value^a^^,^^b^ (cfu)	
*Y*. *pestis* strain	s.c. route	i.n. route
Kimberley53	1–3	2,0 x10^3^
Kim53Δ*nlpD*	>2×10^7^	>4×10^7^
Kim53Δ*tatA*	>1×10^7^	>7×10^6^
Kim53Δ*tatC*	>5×10^6^	>5×10^6^
Kim53Δ*tatA ptatA*	<4	<4,4x10^3^
Kim53Δ*tatC ptatC*	<6	<4,8 x10^3^
Kim53Δ*amiC*	<3	<4,5 x10^3^

^a^The “<” symbol indicates that the calculated LD_50_ value is the minimal infection dose tested, under which more than 50 percent of the animals died.

^b^The “>” symbol indicates that the calculated LD_50_ value is the maximal infection dose tested under which less than 50 percent of the animals died.

Of note, similar results to those pertaining to the TatA mutation could be obtained upon deletion of the *tatC* gene, including full restauration of the wild-type phenotype upon trans-complementation (morphology, Fig 5A, growth under iron deprivation, Fig 5B and virulence in the plague murine models, Table 3).

Taken together the data support the notion that the virulence attenuation characterizing the NlpD mutant phenotype maybe attributed to the dysfunctionality of the Tat system via a possible involvement in iron exploitation. This general possible explanation is compatible both with the micro-array transcriptomic data and the direct inspection of the Tat mutants.

## Discussion

The lipoprotein NlpD emerged in a previous functional screen of *Y*. *pestis* genome as an important factor for the manifestation of *Y*. *pestis* virulence. The screen evidenced that NlpD gene disruption is incompatible with the survival of the bacteria in the host during infection [15,20]. In the current report, to gain further insight into the mechanisms underlying the role of NlpD in *Y*. *pestis* pathogenicity, the transcriptomes of the wild type Kim53 strain and the *nlpD* mutant were compared. Considering that the NlpD-mutated bacteria were rapidly cleared from inoculated animals [20], RNA for the transcriptome analysis was collected from bacteria cultured under conditions reminiscent of the initial stages of infection (growth of *Y*. *pestis* cultures at 37°C for several hours).

Examination of the differential transcriptome data clearly indicated that a pronounced membrane stress response is specifically induced in the *nlpD* mutant, as reflected by up-regulation of the *cpxP* and *pspA* genes (S2 Table). The Cpx and Psp systems are membrane stress-response pathways of Gram-negative bacteria that are involved in maintaining the homeostasis of the cytoplasmic membrane [43,44,67]. These systems sense and respond to periplasmic or cytoplasmic protein misfolding that disturb the integrity of the cytoplasmic membrane and could reduce the energy status of the cell [67,68,69,70]. In *Y*. *enterocolitica*, the Psp system has been shown to be essential for protecting bacterial cells against membrane damage due to miss-localization of the T3SS component secretin (YscC) that is induced at the mammalian body temperature of 37°C [71,72,73]. In the present study, *Y*. *pestis* cultures were grown at 37°C for five hours, resulting in induction of the T3SS [16]. However, inspection of the transcriptome data indicated that the expression of secretin was not differentially regulated in the *nlpD* mutant. The expression of only two T3SS components was altered in the *nlpD* mutant (S2 Table), the YscB chaperon that is required for the calcium-dependent regulation of Yop secretion [74] and YopQ (also known as YopK), which plays an important role in the regulation of Yops translocation [75].

The robust induction of Cpx and Psp stress response systems in the *nlpD* mutant suggests that in the absence of NlpD the integrity and/or stability of the membranes are affected and there is an increase in the accumulation of misfolded proteins in the periplasm. Interestingly, NlpE, which is another outer-membrane lipoprotein, is an accessory protein of the Cpx pathway in *E*. *coli* [76]. However, *nlpE* expression is not altered in the *nlpD* mutant compared with the wild-type strain. One may speculate that NlpD plays a similar role in responding to harmful changes that occur in bacterial membranes following exposure to environmental stress. Similarly, it has recently been demonstrated that changes in the peptidoglycan structure are part of the Cpx-mediated adaptation to envelope stress [77]. The proposed involvement of NlpD in the response to extracytoplasmic stress conditions is compatible with the genomic localization of the *nlpD* gene within a genomic stress response locus from which the SurE, Pcm and RpoS proteins are expressed [20].

Many of the differentially modulated pathways in the *nlpD* mutant were related to iron metabolism (~20%, S2 and S3 Tables), suggesting that the acquisition and consumption of iron may have been perturbed by NlpD deletion. The hypothesis that the mutated cells have an impaired ability to exploit iron was further confirmed by the observation that the mutated bacterial cells failed to grow under iron-limiting conditions (Fig 1A). Lowering the levels of free iron is an important host defense strategy that restricts the proliferation of infectious bacteria [78,79], and many pathogens have evolved sophisticated mechanisms to overcome this restriction and maximize the host iron supply [78,80]. Accordingly, we have recently shown that the *Y*. *pestis* EV76 live vaccine protected mice against an immediate lethal challenge with a virulent *Y*. *pestis* strain and that protection was associated with induction of the host heme- and iron-binding proteins hemopexin and transferrin [81].

Close inspection and integration of all the observed phenotypic characteristics of the *Y*. *pestis nlpD* mutant, namely, chain-forming morphology, attenuation of virulence, reduced tolerance to acidic stress, defective iron acquisition and envelope stresses, suggested a striking resemblance to the phenotypic characteristics of bacterial Tat mutants in other pathogens [52,53]. Importantly, similar to the situation of the *Y*. *pestis nlpD* mutant, the loss of virulence of *Yersinia* Tat mutated strains could not be explained by the dysfunction of the T3SS [60,61].

The assumption that the Tat system is impaired in the *nlpD* mutant was supported by the observed decrease in the expression of several known iron-sulfur protein substrates of this system (S2 Table). In addition, the modulation of type VI secretion system genes observed in the *nlpD* mutant (S2 Table) was also observed in Tat mutants of the phylogenetically related strain *Y*. *pseudotuberculosis* [82], and the fish pathogen *E*. *tarda* [83]. Furthermore, although *Y*. *pestis* is a non-motile bacterium, a flagellar operon was modulated in the *nlpD* mutant (S2 Table), suggesting a possible control on motility that characterize Tat mutants in many bacterial pathogens including *Y*. *pseudotuberculosis* [52,59,60]. The assumption that the phenotype of the NlpD mutant is related to deregulation of the Tat system was directly interrogated. Assessment of the functionality of the Tat system in the *nlpD* mutant was performed by visualization (using microscope analysis and confirmed by subcellular fractionation analysis) of the localization of two different Tat reporter substrates: a GFP-reporter protein fused to the signal peptide of the Tat-substrate TorA or the Tat-substrate NapG [25,60,62]. As hypothesized, the Tat system was not functional in the *Y*. *pestis nlpD* mutant (Fig 2), whereas other translocation systems including the inner-membrane embedded Sec pathway (Fig 4), and the T3SS that traverse the inner and outer membrane of the cell [20], were operational in the *nlpD* mutant indistinguishably from the wild-type strain. These observations suggested that inactivation of the Tat system in the *nlpD* mutant did not result from a general destabilization of the bacterial membrane and substantiate the specificity of the phenotype exhibited by the *nlpD* mutant strain.

TatC protein levels (Fig 3B) as well as the membrane localization of this protein (Fig 3C) confirmed that the Tat system is affected in the *nlpD* mutant. The TatC protein level in cell lysates of the *nlpD* mutant was decreased in comparison to the wild-type strain, and this protein was not detected in the mutant cytoplasmic membrane. Since the RNA levels of the Tat genes were similar in wild-type and the *nlpD* mutant, one may speculate that dysfunction of the Tat system in the *nlpD* mutant could have resulted from post-transcriptional molecular events. In *Y*. *pseudotuberculosis*, a *tatC* mutant was highly attenuated for virulence following oral or intraperitoneal infections [60]. The system was important for different virulence-related stress responses as well as for iron uptake [60]. Additional studies have indicated that the loss of virulence is related to the SufI Tat-substrate that was found to be required for establishment of systemic infection [84].

The *Y*. *pestis tatA* and *tatC* mutants were avirulent in mice. In *Y*. *pestis* CO92, the *tatA* mutant was highly attenuated in the bubonic and aerosol-infection mouse model but to a lesser extent in the intranasal infection model [61]. Attenuation of virulence in the bubonic plague model is therefore similar in both *Y*. *pestis tatA* mutant strains. The differences between the virulence characteristics of the Kim53 and CO92 *tatA* mutants in the i.n. infection model may reflect variations in the mouse strain used for the evaluation of virulence or the genetic diversity between the two *Y*. *pestis* strains.

The known functions of the NlpD lipoprotein, which belong to the M23-LytM endopeptidase family, involves the regulation of peptidoglycan hydrolysis and cell morphogenesis [20,21,85,86]. In *E*. *coli*, the NlpD protein is not catalytically active but controls the activity and recruitment to the septum of the cell wall amidase—AmiC, which is a known Tat system substrate [85,87,88,89]. Interestingly, the *Y*. *pestis amiC* mutant retained the wild-type single cell morphology (S7 Fig) and virulence characteristics (Table 3). These observations suggest that in *Y*. *pestis* the mode of interactions between NlpD and AmiC maybe different than in *E*. *coli*.

The present study shows that the *nlpD* mutant exhibited impaired Tat activity as well as limited iron acquisition. Both of these characteristics may have represented the cause for the severe virulence attenuation exhibited by the *nlpD* mutant. Considering the similarity between the phenotypic characteristics of the *nlpD* mutant and the *tat* mutants including chain morphology, iron assimilation defect and loss of virulence, the present data suggest a novel link between NlpD and the Tat system affecting *Y*. *pestis* pathogenesis.

The molecular mechanisms underlying the possible relationships between NlpD, the Tat system components and iron assimilation remain to be deciphered and raise several questions including the possible interactions between the outer membrane-predicted NlpD lipoprotein, and iron assimilation systems or the inner membrane Tat proteins. Studies addressing some of these issues are currently underway in our laboratory.

## Supporting information

S1 Fig Expression of TatC, NlpD and Pcm in *Y. pestis* tat mutants. A. Coomassie blue stain (upper panel) and Western blot analysis of TatC and NlpD protein levels in total cell lysates (lower panel). Cultures of *Y*. *pestis* strains were inoculated (initial OD_660_ = 0.01) and incubated for an additional 24 hours at 37°C. Western blot analysis was performed with anti-NlpD, anti-TatC and anti-Pcm antibodies, to equal amount of cells/lane. The Pcm protein served as loading control. Coomassie blue stained gel and the blots were derived from the same experiment and were processed in parallel. B. The original uncropped Western blots depicted in A. The portions of the Western blots used in panel A, are indicated by black rectangles.
(TIF)

S2 Fig Differentially expressed genes in *Y. pestis nlpD* mutant relative to the parental Kim53 strain. A graphical presentation of the fold changes of up-regulated (A) or down-regulate (B) genes in the *nlpD* mutant relative to the wild-type Kim53 strain. The genes are categorized according to their functional classification (see S2, S3, S4 and S5 Tables).
(TIF)

S3 Fig Comparison of transcription measurements by microarray and real-time PCR assays. The relative transcriptional levels of the genes were determined by real-time RT-PCR. The log2 values were plotted against the microarray data log2 values. The correlation coefficient (R^2^) for comparison of the two datasets is 0.8459.
(TIF)

S4 Fig Intracellular localization of the NapG-GFP Tat-reporter protein in *Y. pestis* strains. *Y*. *pestis* strains: Kim53p:*napG*:GFP, Kim53Δ*nlpD*:*napG*:GFP and Kim53Δ*nlpD*+*pnlpD*:*napG*:GFP were inspected under a fluorescence microscope for identification of NapG-GFP (Tat substrate protein fused to GFP) localization. The scale bar represents 1 μm.
(TIF)

S5 Fig Original uncropped Western blots depicted in Fig 2B. See legend to Fig 2. The original uncropped Western blots depicted in Fig 2B. The portions of the Western blots used are indicated by black rectangles. *The *tatB* mutant is not presented in Fig 2 due to a polar effect caused by *tatB* deletion that tampered TatC expression.
(TIF)

S6 Fig Original uncropped Western blots depicted in Fig 3B. See legend to Fig 3. The portions of the coomassie stained gel and the Western blots used are indicated by black rectangles.
(TIF)

S7 Fig Phenotypic characterization of *Y. pestis* Kim53Δ*amiC* strain. Gram staining of *Y*. *pestis* Δ*amiC* mutant was performed. Bacilli were observed by light microscopy at a magnification of ×1000. Scale bar = 10 μm.
(TIF)

S1 Table Primers used in this study. (DOCX)

S2 Table Genes up-regulated in *Y. pestis nlpD* mutant relative to the parental Kim53 strain. (DOCX)

S3 Table Genes down-regulated in *Y. pestis nlpD* mutant relative to the parental Kim53 strain. (DOCX)

S4 Table Excel file specifying the up-regulated YPO gene numbers. (XLSX)

S5 Table Excel file specifying the down-regulated YPO gene numbers. (XLSX)

S6 Table qRT-PCR-determined differentially expressed genes. (DOCX)
