# Supplementary figures and images for: Disruption of the NlpD lipoprotein of the plague pathogen Yersinia pestis affects iron acquisition and the activity of the twin-arginine translocation system
Source: PLoS Negl Trop Dis. 2019 Jun 6;13(6):e0007449. doi: 10.1371/journal.pntd.0007449 (PMC6553720; doi:10.1371/journal.pntd.0007449)

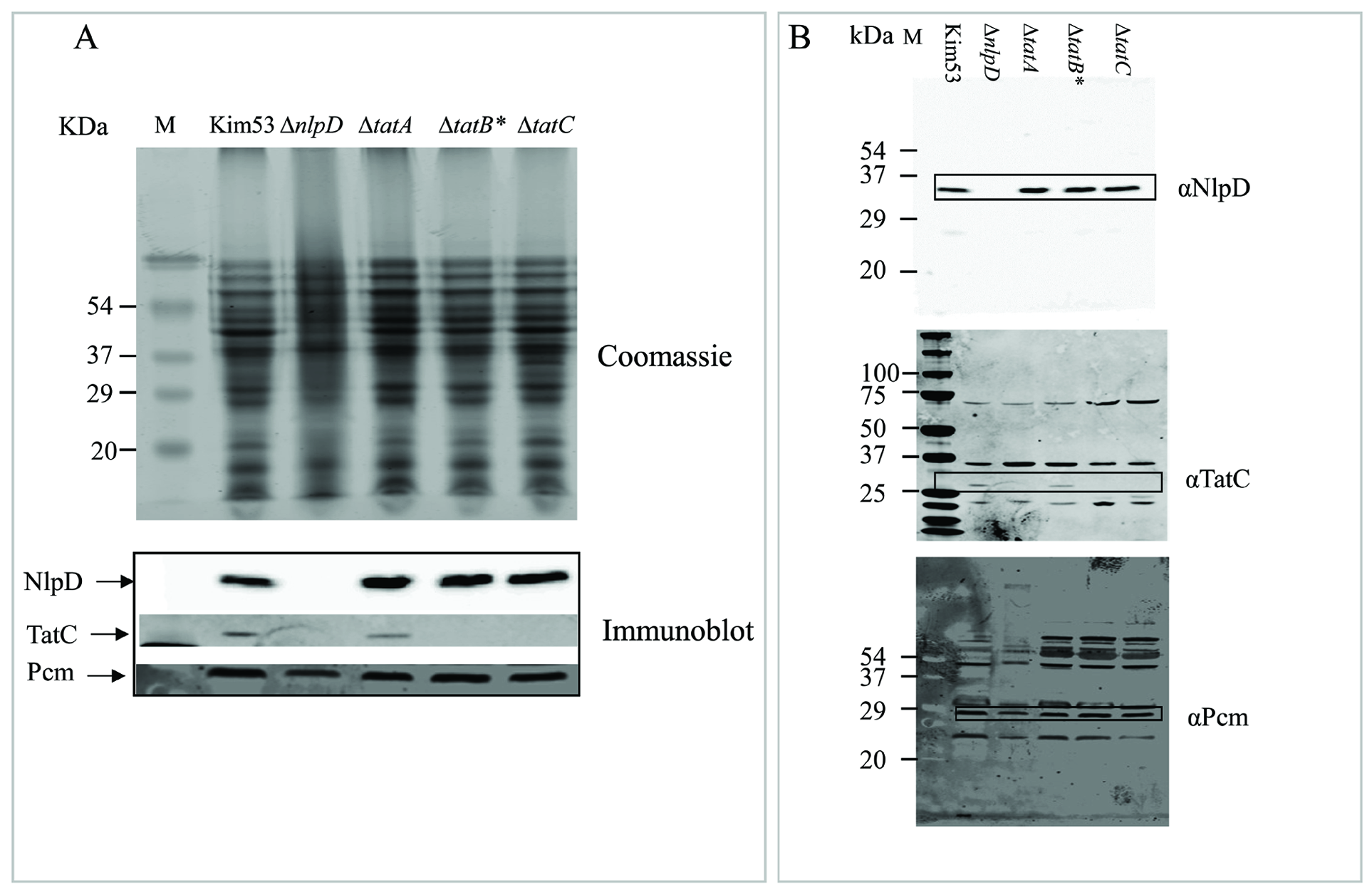

Supplement: S1 Fig — A. Coomassie blue stain (upper panel) and Western blot analysis of TatC and NlpD protein levels in total cell lysates (lower panel). Cultures of Y. pestis strains were inoculated (initial OD660 = 0.01) and incubated for an additional 24 hours at 37°C. Western blot analysis was performed with anti-NlpD, anti-TatC and anti-Pcm antibodies, to equal amount of cells/lane. The Pcm protein served as loading control. Coomassie blue stained gel and the blots were derived from the same experiment and were processed in parallel. B. The original uncropped Western blots depicted in A. The portions of the Western blots used in panel A, are indicated by black rectangles. (TIF) [file pntd.0007449.s001.tif]

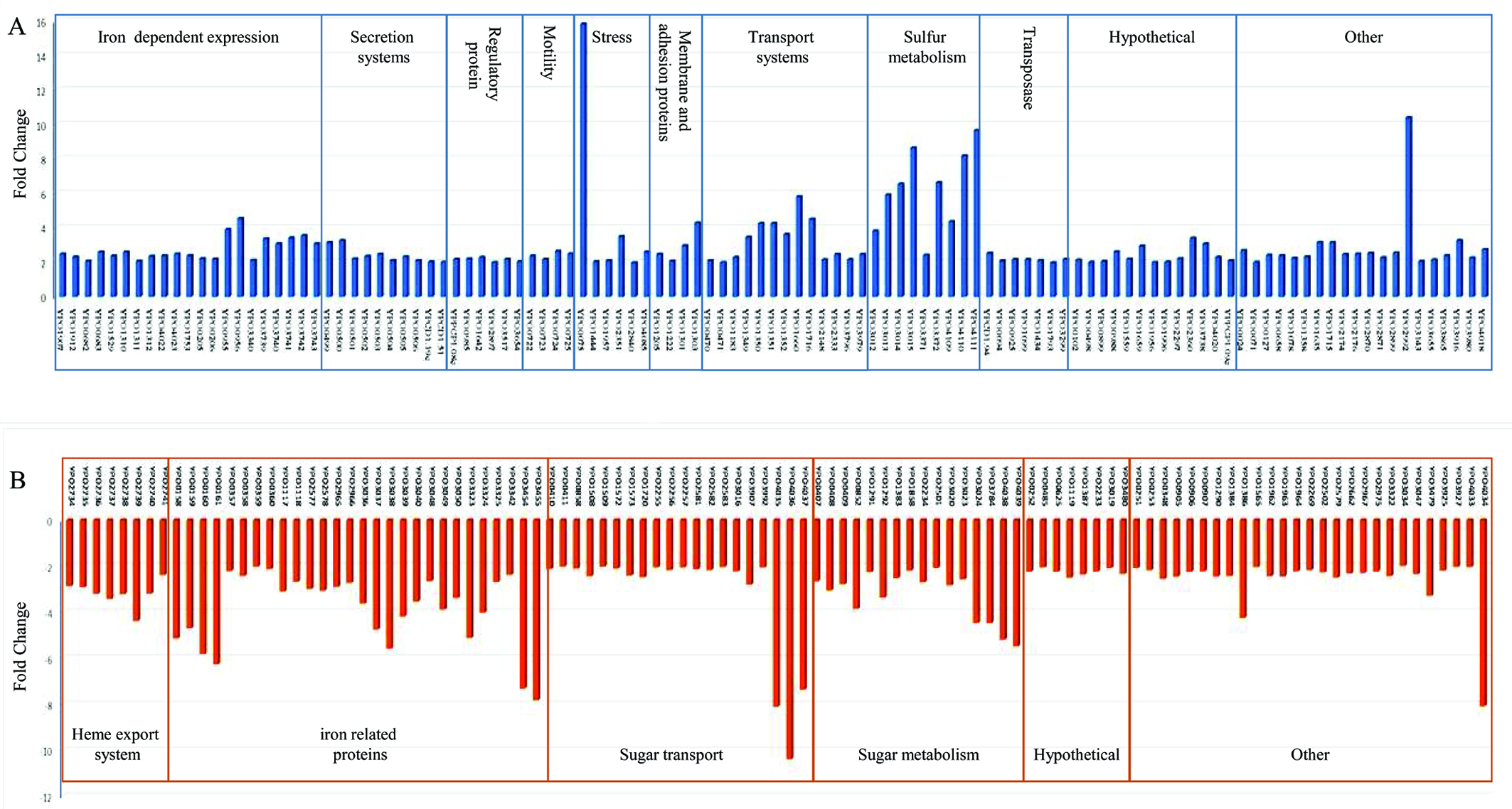

Supplement: S2 Fig — A graphical presentation of the fold changes of up-regulated (A) or down-regulate (B) genes in the nlpD mutant relative to the wild-type Kim53 strain. The genes are categorized according to their functional classification (see S2, S3, S4 and S5 Tables). (TIF) [file pntd.0007449.s002.tif]

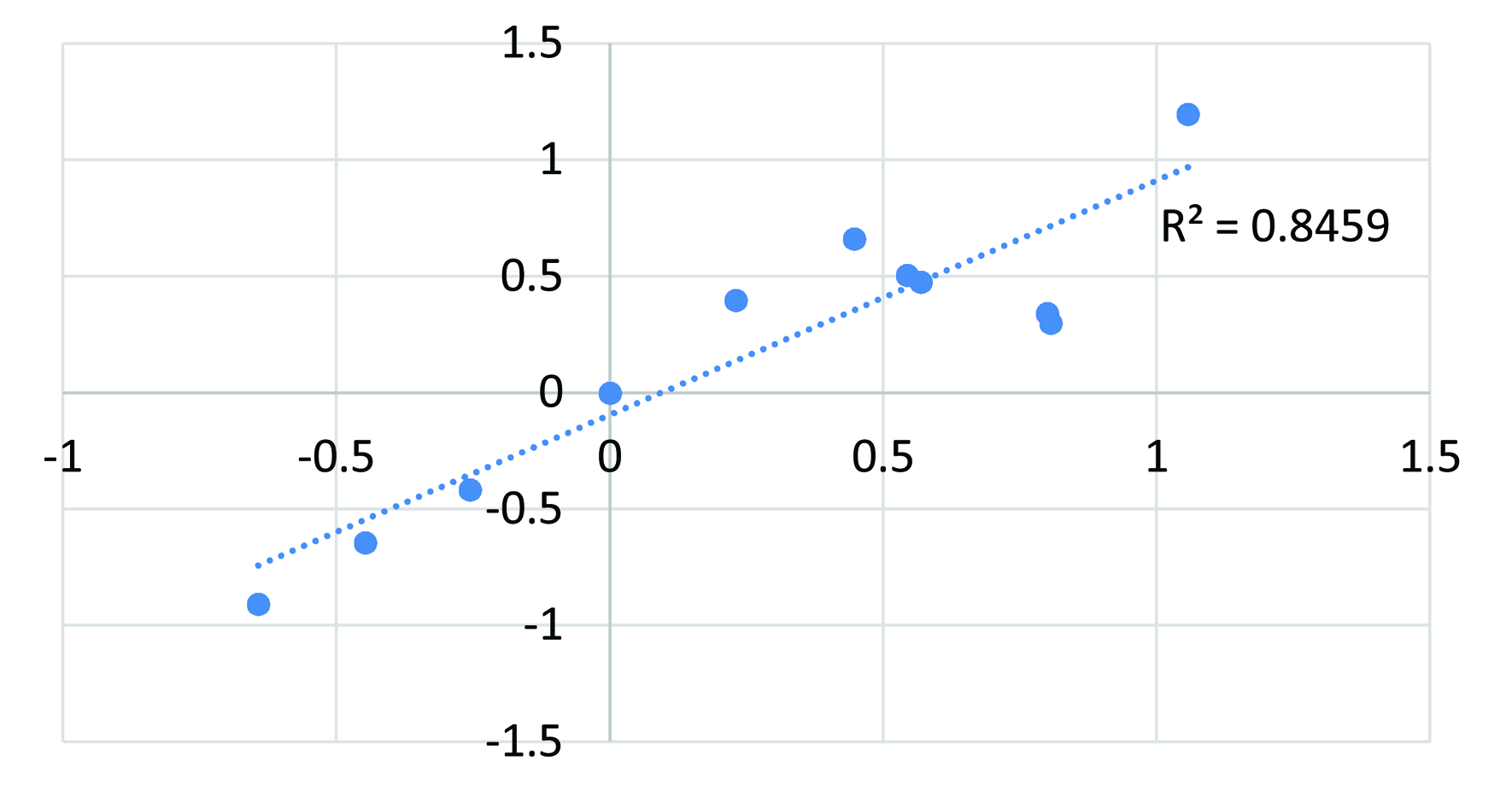

Supplement: S3 Fig — The relative transcriptional levels of the genes were determined by real-time RT-PCR. The log2 values were plotted against the microarray data log2 values. The correlation coefficient (R2) for comparison of the two datasets is 0.8459. (TIF) [file pntd.0007449.s003.tif]

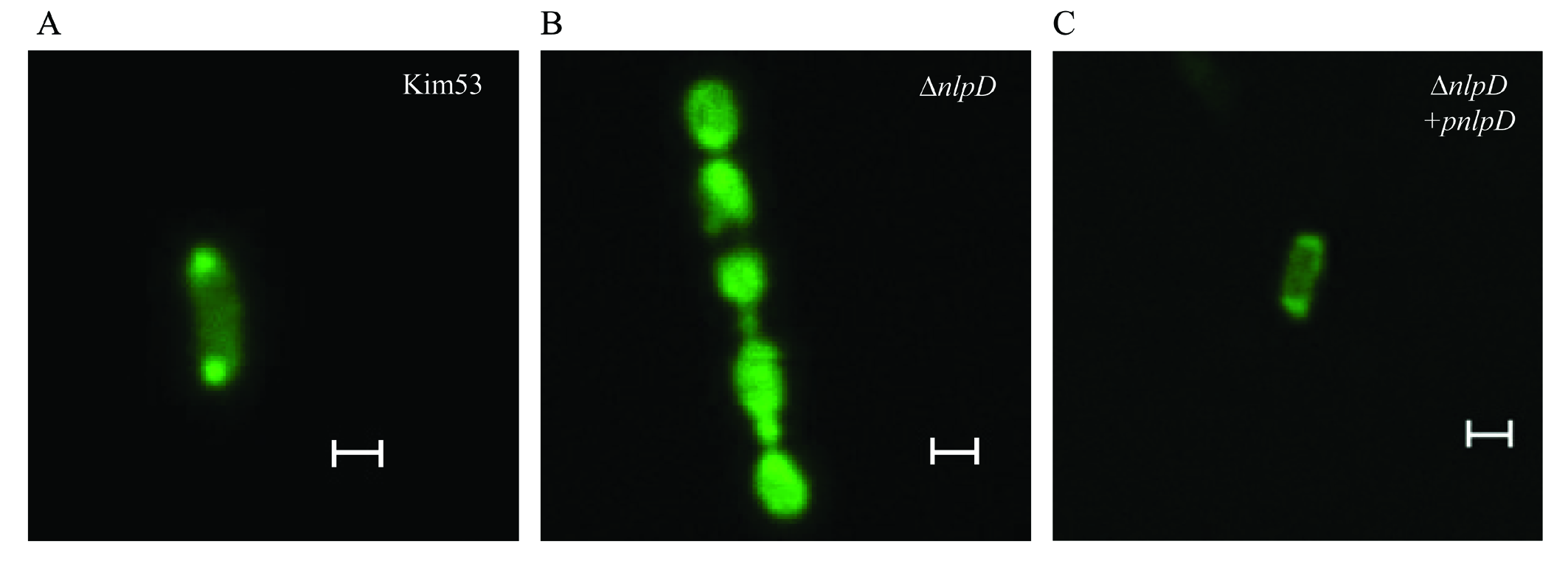

Supplement: S4 Fig — Y. pestis strains: Kim53p:napG:GFP, Kim53ΔnlpD:napG:GFP and Kim53ΔnlpD+pnlpD:napG:GFP were inspected under a fluorescence microscope for identification of NapG-GFP (Tat substrate protein fused to GFP) localization. The scale bar represents 1 μm. (TIF) [file pntd.0007449.s004.tif]

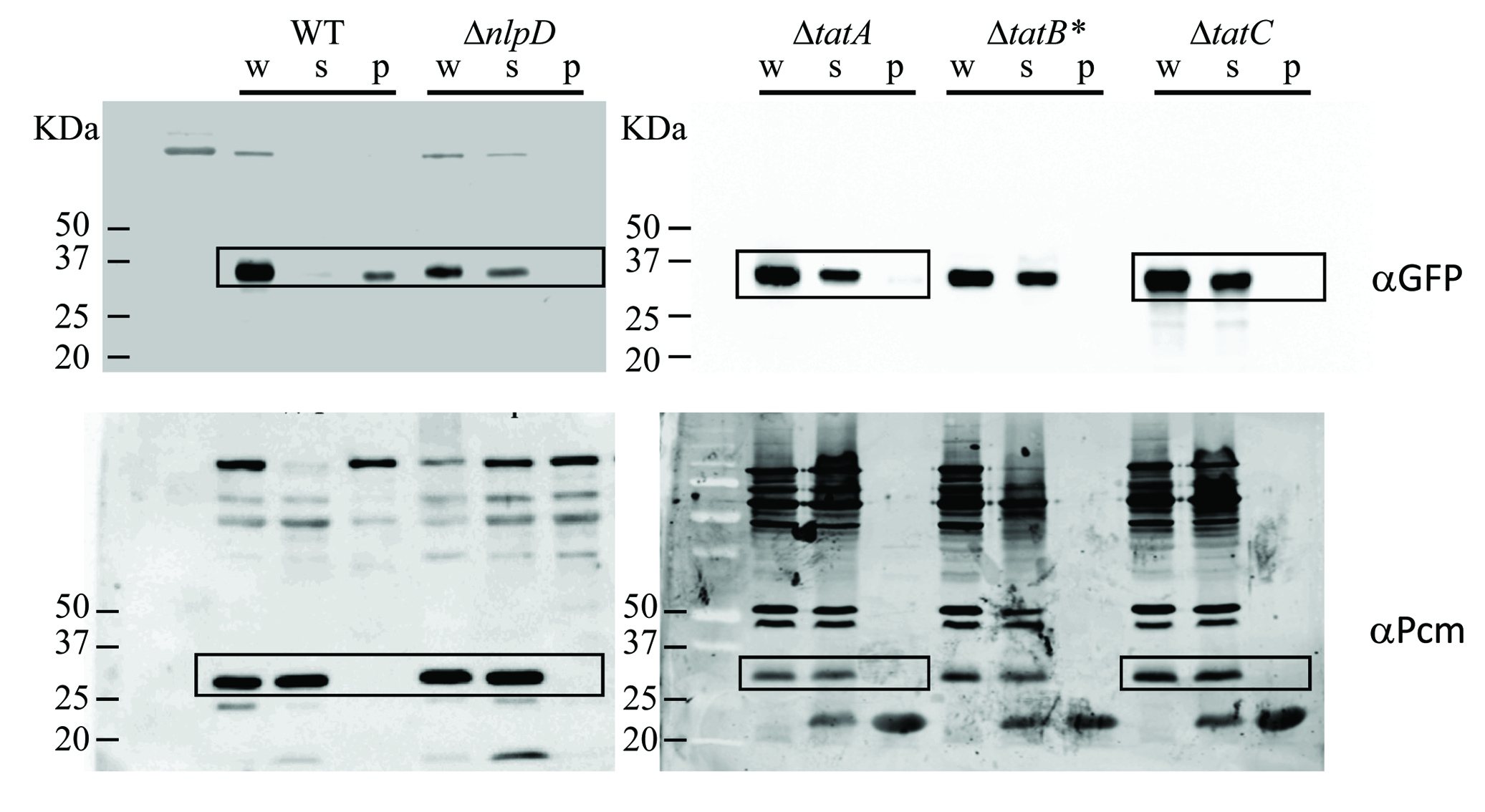

Supplement: S5 Fig — The original uncropped Western blots depicted in Fig 2B. The portions of the Western blots used are indicated by black rectangles. *The tatB mutant is not presented in Fig 2 due to a polar effect caused by tatB deletion that tampered TatC expression. (TIF) [file pntd.0007449.s005.tif]

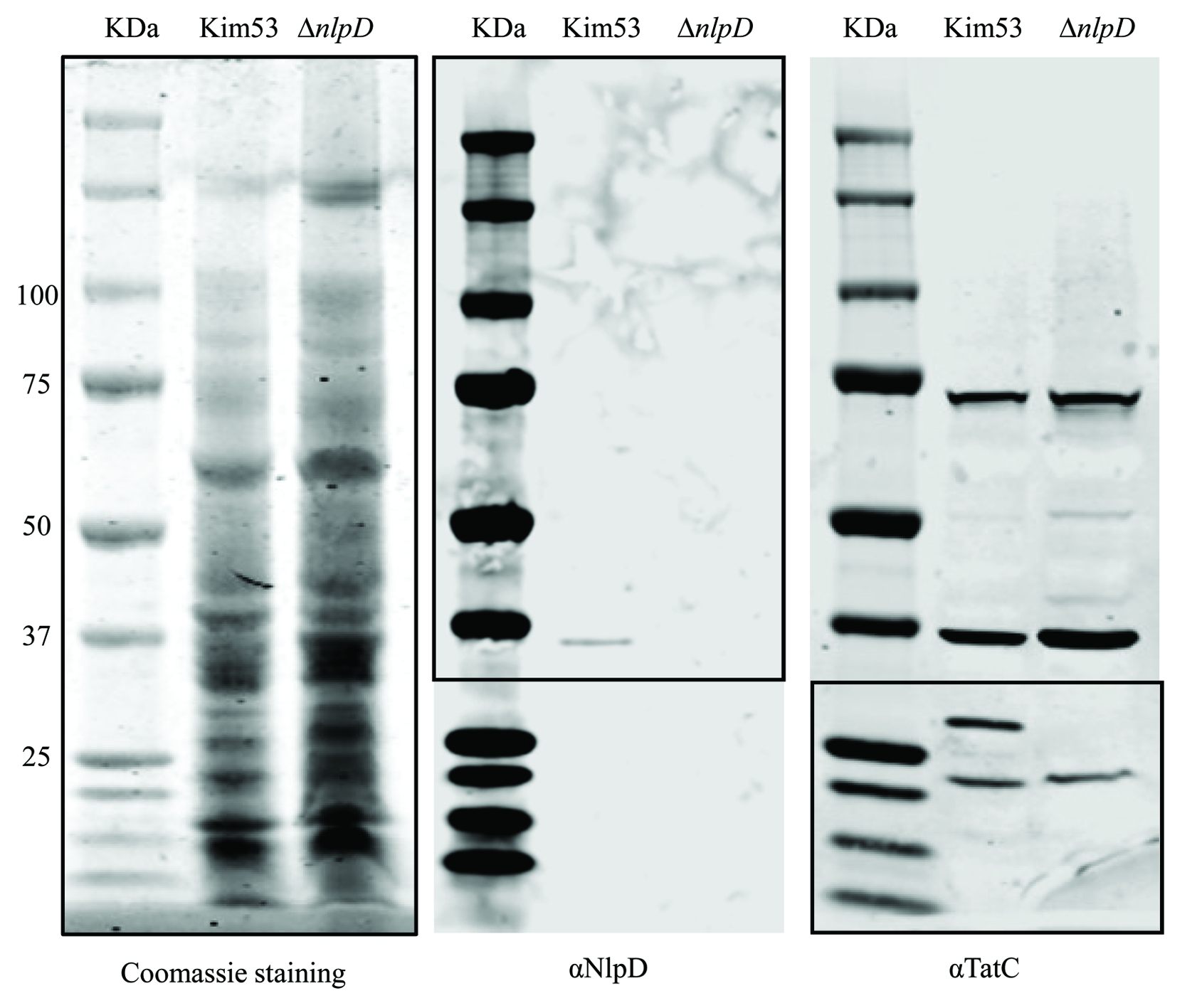

Supplement: S6 Fig — The portions of the coomassie stained gel and the Western blots used are indicated by black rectangles. (TIF) [file pntd.0007449.s006.tif]

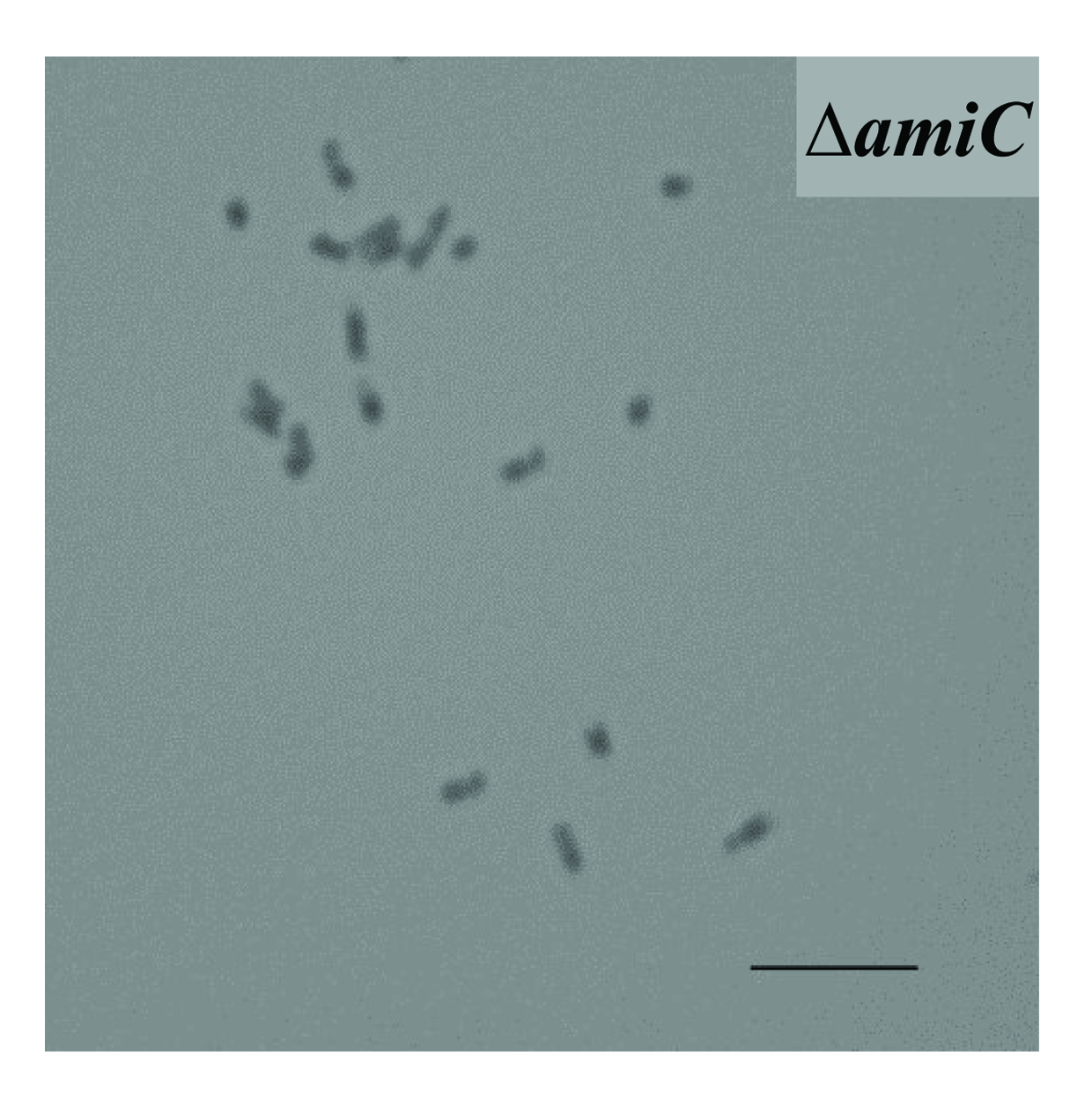

Supplement: S7 Fig — Gram staining of Y. pestis ΔamiC mutant was performed. Bacilli were observed by light microscopy at a magnification of ×1000. Scale bar = 10 μm. (TIF) [file pntd.0007449.s007.tif]
