# Supplementary material for: Disruption of the NlpD lipoprotein of the plague pathogen Yersinia pestis affects iron acquisition and the activity of the twin-arginine translocation system
Source: PLoS Negl Trop Dis. 2019 Jun 6;13(6):e0007449. doi: 10.1371/journal.pntd.0007449 (PMC6553720; doi:10.1371/journal.pntd.0007449)
Supplement: S1 Table — (DOCX) [file pntd.0007449.s008.docx]

Table S1 Primers used in this study

| qRT-PCR | | | | | | |
| --- | --- | --- | --- | --- | --- | --- |
| PID | Gene  Name | | | Forward Primer | | Reverse Primer |
| YPO0075 | *cpxP* | | | CCTTTGCTGCCGACAACACT | | GCATCAAGTCACGCATTTGC |
| YPO0723 | *flgC* | | | ATGAGCCCAATCACCCAATG | | TCTGCTGCATGCTTTTCACACT |
| YPO0956 | YPO0956 | | | ATACCGATGCACGCCTGACT | | GTGCGCCAATACCTGCTTTC |
| YPO1045 | *tsf* | | | AAAGATGCTGGCTTCAAAGCA | | ACGGCGAATGTTGATGTTCTC |
| YPO1301 | *psaE* | | | GCTGCTGTTAGAGTGTCTTTATTTACG | | TCCGGGTTATTTTACGGAATACA |
| YPO1302 | *psaF* | | | CCCTGATAATATGGCCAGAGGAT | | AAGTGGATCGTTTCTCCATTGAC |
| YPO1415 | *pyrD* | | | TCCAGAACGTGCCCATGAAT | | AGGCCCGCAGCCAAAC |
| YPO1715 | *ybjR* | | | CCCACTATCCCTCTGTTGCAA | | CACCAGCTTTCTTTGGGTGAGT |
| YPO1907 | *irp5* | | | GTACCCTGCAACAGGTTTTCG | | CGACGTCGTTCTCGTCTTGA |
| YPO2739 | *ccmF* | | | TCGCGTTTGCTTTTGCAAT | | CCCCACCCCAATTCATAATAAG |
| YPO3355 | *appR* | | | TACGCACAGCACGAGAGCTT | | CGCCGCCTAAAGGTGTATCA |
| YPO3356 | *nlpD* | | | TTCTCGTGGGCAACCTATTCTC | | TCCCGGACCAGCATTGTATC |
| YPO3776 | *tatC* | | | AGCGCCAGAAAGCGTACTGA | | TTCAATGCTTCTGGTGTTGTTACA |
| YPO4034 | YPO4034 | | | CAAACAGACCCGCCCTTACA | | TTCAACTTTGATCCCCTTGCA |
|  | | | | | | |
| Complementation of *tat* genes expression | | | | | | |
| PID | | Gene  Name | Primer name | | Primer sequence | |
| YPO3778 | | tatA | pWKS-*tatA*-For | | CTCGAGGTCGACGGTATCGATAAGCTTACGAGGTAACTAATGGGCAGTATCGGTTGGGCAC | |
| YPO3778 | | tatA | pWKS-*tatA*-Rev | | CGGGGCGGCGCTCTAGATGCCTCAGCCGGATCTCATCCCTGTTCTTTTTCGTGACTC | |
| YPO3776 | | tatC | pWKS-*tatC*-For | | CTCGAGGTCGACGGTATCGATAAGCTTACGAGGTAACTAATGGCTGTTGATGATACCC | |
| YPO3776 | | tatC | pWKS-*tatC*-Rev | | CGGGGCGGCGCTCTAGATGCCTCAGCCGGATCTCATTAAGGTGCTTTCGGATGG | |
