# Supplementary material for: Disruption of the NlpD lipoprotein of the plague pathogen Yersinia pestis affects iron acquisition and the activity of the twin-arginine translocation system
Source: PLoS Negl Trop Dis. 2019 Jun 6;13(6):e0007449. doi: 10.1371/journal.pntd.0007449 (PMC6553720; doi:10.1371/journal.pntd.0007449)
Supplement: S2 Table — (DOCX) [file pntd.0007449.s009.docx]

Table S2 Genes up-regulated in *Y. pestis* *nlpD* mutant relative to the parental Kim53 strain

| **Gene ID** | **Gene name** | | **Annotation^1^** | **Median FC^2^** |
| --- | --- | --- | --- | --- |
| **Iron dependent expression** | | | |  |
| Ybt siderophore iron-transport system | | | |  |
| YPO1907 | irp5 | | yersiniabactin siderophore biosynthetic protein | 2.5 |
| YPO1912 | ybtA | | transcriptional regulator YbtA | 2.33 |
| ExbBD-TonB complex | | |  |  |
| YPO0682 |  | | MotA/TolQ/ExbB proton channel family protein | 2.08 |
| YPO0683 |  | | biopolymer transport protein ExbD | 2.61 |
| Putative Ysu siderophore iron-transport system | | | |  |
| YPO1529 |  | | decarboxylase | 2.38 |
| Putative Yiu iron-ABC transporter | | | |  |
| YPO1310 |  | | periplasmic substrate-binding transport protein | 2.61 |
| YPO1311 |  | | FecCD-family membrane transport protein | 2.08 |
| YPO1312 |  | | siderophore ABC transporter, ATP-binding subunit | 2.37 |
| Putative fit iron ABC transporter | | | |  |
| YPO4022 |  | | iron transport protein | 2.4 |
| YPO4023 |  | | iron transport permease | 2.5 |
| Ferric iron-transport system | | | |  |
| YPO1753 | fcuA | | ferrichrome receptor protein | 2.41 |
| Iron storage |  | |  |  |
| YPO0205 | bfd | | bacterioferritin-associated ferredoxin | 2.23 |
| YPO0206 | bfr | | bacterioferritin | 2.21 |
| Other iron related genes | | | |  |
| YPO0955 |  | | hypothetical protein/iron related* [[1](#_ENREF_1)] | 3.91 |
| YPO0956 |  | | hydroxamate-type ferrisiderophore receptor | 4.56 |
| YPO3340 |  | | Pseudogene/Putative ferric siderophore receptor* [[2](#_ENREF_2),[3](#_ENREF_3)] | 2.14 |
| Thiamine biosynthesis | | |  |  |
| YPO3739 | thiC | | thiamine biosynthesis protein ThiC | 3.38 |
| YPO3740 | thiE | | thiamine-phosphate pyrophosphorylase | 3.09 |
| YPO3741 | thiF | | thiamine biosynthesis protein ThiF | 3.42 |
| YPO3742 | thiG | | thiamine biosynthesis protein ThiG | 3.56 |
| YPO3743 | thiH | | thiamine biosynthesis protein ThiH | 3.1 |
|  | | | |  |
| **Secretion systems** | | | |  |
| Type VI secretion system | |  |  |  |
| YPO0499 |  | | hypothetical protein/TTS6* [[4](#_ENREF_4)] | 3.17 |
| YPO0500 |  | | hypothetical protein/TTS6* [[4](#_ENREF_4)] | 3.28 |
| YPO0501 |  | | hypothetical protein/TTS6* [[4](#_ENREF_4)] | 2.22 |
| YPO0502 |  | | hypothetical protein/TTS6* [[4](#_ENREF_4)] | 2.37 |
| YPO0503 |  | | hypothetical protein/TTS6* [[4](#_ENREF_4)] | 2.49 |
| YPO0504 |  | | hypothetical protein/TTS6* [[4](#_ENREF_4)] | 2.13 |
| YPO0505 |  | | hypothetical protein/TTS6* [[4](#_ENREF_4)] | 2.34 |
| YPO0506 | clpB | | Clp ATPase [[4](#_ENREF_4)] | 2.12 |
|  |  | |  |  |
|  |  | |  |  |
| Type III secretion system | |  |  |  |
| YPCD1.19c | yopQ | | Yop targeting protein | 2.04 |
| YPCD1.51 | yscB | | type III secretion apparatus component | 2.02 |
| **Regulatory protein** | | |  |  |
| YPPCP1.08c |  | | putative transcriptional regulator | 2.19 |
| YPO0985 | yspR | | quorum-sensing transcriptional regulator | 2.22 |
| YPO1642 | cscR | | sucrose operon repressor LacI-family | 2.3 |
| YPO2897 | iscR | | DNA-binding transcriptional regulator IscR | 2.01 |
| YPO3517 | argR | | arginine repressor | 2.2 |
| YPO3654 | fis | | Fis family transcriptional regulator | 2.05 |
| **Motility** |  | |  |  |
| YPO0722 | flgB | | flagellar basal-body rod protein | 2.38 |
| YPO0723 | flgC | | flagellar basal-body rod protein | 2.19 |
| YPO0724 | flgD | | basal-body rod modification protein | 2.65 |
| YPO0725 | flgE | | flagellar hook protein | 2.51 |
| **Stress** |  | |  |  |
| YPO0075 | cpxP | | periplasmic stress adaptor protein CpxP | 15.82 |
| YPO1444 | hspQ | | heat shock protein HspQ | 2.05 |
| YPO1957 | phoH | | putative phosphate starvation-inducible protein | 2.12 |
| YPO2351 | pspA | | phage shock protein A | 3.51 |
| YPO2840 |  | | chaperone | 2 |
| YPO4085 | ibpA | | heat shock protein | 2.62 |
| **Membrane and adhesion proteins** | | | |  |
| YPO1205 | ompC2 | | putative outer membrane porin | 2.49 |
| YPO1222 | ompC | | porin | 2.08 |
| YPO1301 | psaE | | regulatory protein | 2.98 |
| YPO1303 | psaA | | pH 6 antigen precursor (antigen 4) (adhesin) | 4.3 |
| **Transport systems** | | |  |  |
| YPO0470 | nhaA | | pH-dependent sodium/proton antiporter | 2.12 |
| YPO0471 | nhaR | | transcriptional activator protein NhaR | 2.01 |
| YPO1183 |  | | substrate-binding periplasmic transport protein | 2.3 |
| YPO1349 | artM | | arginine transport system permease protein | 3.47 |
| YPO1350 | artQ | | arginine transport system permease protein | 4.27 |
| YPO1351 | artI | | arginine-binding periplasmic protein 1 precursor | 4.27 |
| YPO1352 | artP | | arginine transport ATP-binding protein | 3.63 |
| YPO1660 | mgtC | | Mg(2+) transport ATPase protein C | 5.82 |
| YPO1716 | gltP | | transport protein | 4.52 |
| YPO2148 |  | | multidrug resistance protein | 2.16 |
| YPO2333 | qacE | | quaternary ammonium compound-resistance protein | 2.48 |
| YPO3796 | ugpB | | glycerol-3-phosphate-binding periplasmic protein | 2.17 |
| YPO3979 |  | | GntP family permease | 2.48 |
| **Sulfur metabolism** | | |  |  |
| YPO3012 | cysA | | sulfate transport ATP-binding protein | 3.83 |
| YPO3013 | cysW | | sulfate transport system permease protein CysW | 5.92 |
| YPO3014 | cysT | | sulfate transport system permease protein CysT | 6.55 |
| YPO3015 | cysP | | thiosulfate-binding protein | 8.64 |
| YPO3371 | cysI | | sulfite reductase subunit beta | 2.43 |
| YPO3372 | cysJ | | sulfite reductase subunit alpha | 6.63 |
| YPO4109 |  | | amino acid transport system permease | 4.38 |
| YPO4110 |  | | ABC transporter permease | 8.18 |
| YPO4111 |  | | putative periplasmic solute-binding protein | 9.66 |
| **Transposase** |  | |  |  |
| YPCD1.94 |  | | putative transposase | 2.55 |
| YPO0094 | tnp | | transposase for the IS1541 insertion element | 2.1 |
| YPO0925 | tnp | | transposase for the IS1541 insertion element | 2.18 |
| YPO1099 | tnp | | transposase for the IS1541 insertion element | 2.18 |
| YPO1434 | tnp | | transposase for the IS1541 insertion element | 2.13 |
| YPO1793 | tpn | | Inv; pseudogene | 2 |
| YPO3299 | tnp | | transposase for the IS1541 insertion element | 2.19 |
| **Hypothetical** |  | |  |  |
| YPO0102 |  | | hypothetical protein | 2.15 |
| YPO0498 |  | | hypothetical protein | 2.02 |
| YPO0899 |  | | hypothetical protein | 2.07 |
| YPO0988 |  | | hypothetical protein | 2.63 |
| YPO1559 |  | | hypothetical protein | 2.21 |
| YPO1659 |  | | hypothetical protein | 2.96 |
| YPO1956 |  | | hypothetical protein | 2.01 |
| YPO1996 |  | | hypothetical protein | 2.03 |
| YPO2297 |  | | hypothetical protein | 2.23 |
| YPO2360 |  | | hypothetical protein | 3.41 |
| YPO3738 |  | | hypothetical protein | 3.08 |
| YPO4020 |  | | hypothetical protein | 2.31 |
| YPPCP1.09c |  | | hypothetical protein | 2.12 |
| **Other** |  | |  |  |
| YPO0024 | glnA | | glutamine synthetase | 2.7 |
| YPO0071 |  | | methyltransferase | 2.02 |
| YPO0127 |  | | DNA uptake protein | 2.43 |
| YPO0658 | ribB | | 3,4-dihydroxy-2-butanone 4-phosphate synthase | 2.41 |
| YPO1078 | mltD | | membrane-bound lytic murein transglycosylase D | 2.26 |
| YPO1358 | poxB | | pyruvate dehydrogenase | 2.34 |
| YPO1635 |  | | lipoprotein | 3.18 |
| YPO1715 | ybjR | | N-acetylmuramoyl-L-alanine amidase | 3.17 |
| YPO2174 |  | | nucleotide sugar dehydrogenase | 2.48 |
| YPO2176 | tdk | | thymidine kinase | 2.5 |
| YPO2870 | guaA | | GMP synthase | 2.55 |
| YPO2871 | guaB | | inosine-5'-monophosphate dehydrogenase | 2.29 |
| YPO2899 | suhB | | inositol monophosphatase | 2.56 |
| YPO2992 | cysK (cysZ) | | cysteine synthase A | 10.41 |
| YPO3143 | glnK | | nitrogen regulatory protein P-II | 2.07 |
| YPO3655 |  | | tRNA-dihydrouridine synthase B | 2.16 |
| YPO3865 | wzzE | | lipopolysaccharide biosynthesis protein | 2.41 |
| YPO3916 |  | | peroxiredoxin/glutaredoxin family protein | 3.28 |
| YPO3980 | glxK | | glycerate kinase | 2.28 |
| YPO4018 | cysM | | pyridoxal-phosphate dependent protein | 2.75 |

^1^Gene name and annotation is according to *Y. pestis* CO92 strain (NC_003143.1).

The annotation was inferred from *Y. pestis* CO92 genome sequencing project and was complemented by annotations from orthologous sequences of *Y. pestis* KIM strain (NC_004088.1) and *E. coli* K12 (NC_000913) (Expectation value < e^-10^ and alignment over 80 % of the protein sequence).

Additional annotation data was based on the KEGG website (<http://www.genome.jp/kegg/>) and the Transporter Proteins database of *Yersinia pestis* CO92 (<http://www.membranetransport.org/transporter2.php?oOID=ypes1> ; (*) a novel/updated annotation available from recent publications is also provided.

^2^ FC-fold changes.

1. Gao H, Zhou D, Li Y, Guo Z, Han Y, et al. (2008) The iron-responsive Fur regulon in Yersinia pestis. J Bacteriol 190: 3063-3075.

2. Sebbane F, Lemaitre N, Sturdevant DE, Rebeil R, Virtaneva K, et al. (2006) Adaptive response of Yersinia pestis to extracellular effectors of innate immunity during bubonic plague. Proc Natl Acad Sci U S A 103: 11766-11771.

3. Han Y, Qiu J, Guo Z, Gao H, Song Y, et al. (2007) Comparative transcriptomics in Yersinia pestis: a global view of environmental modulation of gene expression. BMC Microbiol 7: 96.

4. Robinson JB, Telepnev MV, Zudina IV, Bouyer D, Montenieri JA, et al. (2009) Evaluation of a Yersinia pestis mutant impaired in a thermoregulated type VI-like secretion system in flea, macrophage and murine models. Microb Pathog 47: 243-251.
