# Supplementary material for: Disruption of the NlpD lipoprotein of the plague pathogen Yersinia pestis affects iron acquisition and the activity of the twin-arginine translocation system
Source: PLoS Negl Trop Dis. 2019 Jun 6;13(6):e0007449. doi: 10.1371/journal.pntd.0007449 (PMC6553720; doi:10.1371/journal.pntd.0007449)
Supplement: S3 Table — (DOCX) [file pntd.0007449.s010.docx]

Table S3 Genes down-regulated in *Y. pestis* *nlpD* mutant relative to the parental Kim53 strain

| **Gene ID** | **Gene name** | | **Annotation^1^** | **Median FC ^2^** |
| --- | --- | --- | --- | --- |
| **Heme export system** | | | |  |
| YPO2734 | ccmA | cytochrome c biogenesis protein CcmA | | -2.87 |
| YPO2735 | ccmB | heme exporter protein B | | -2.92 |
| YPO2736 | ccmC | heme exporter protein C | | -3.21 |
| YPO2737 | ccmD | heme exporter protein D | | -3.43 |
| YPO2738 | ccmE | cytochrome c-type biogenesis protein CcmE | | -3.22 |
| YPO2739 | ccmF | cytochrome c-type biogenesis protein | | -4.39 |
| YPO2740 | ccmG | thiol:disulfide interchange protein DsbE | | -3.20 |
| YPO2741 | ccmH | cytochrome c-type biogenesis protein | | -2.39 |
| **iron related proteins** ( iron-sulfur proteins and associated proteins, iron-dependent expression) | | | |  |
| YPO0158 | cysG | siroheme synthase | | -5.14 |
| YPO0159 | nirC | nitrite transporter NirC | | -4.71 |
| YPO0160 | nirD | nitrite reductase small subunit | | -5.83 |
| YPO0161 | nirB | nitrite reductase | | -6.27 |
| YPO0357 | frdD | fumarate reductase subunit D | | -2.22 |
| YPO0358 | frdC | fumarate reductase subunit C | | -2.43 |
| YPO0359 | frdB | fumarate reductase iron-sulfur protein | | -2.03 |
| YPO0360 | frdA | fumarate reductase flavoprotein subunit 2 | | -2.13 |
| YPO1117 | cydA | cytochrome D ubiquinol oxidase subunit I | | -3.1 |
| YPO1118 | cydB | cytochrome D ubiquinol oxidase subunit II | | -2.69 |
| YPO2577 |  | aldehyde dehydrogenase | | -3 |
| YPO2578 |  | thiamine pyrophosphate-dependent protein | | -3.07 |
| YPO2965 | dmsA | dimethyl sulfoxide reductase chain A protein | | -2.91 |
| YPO2966 | dmsB | dimethyl sulfoxide reductase chain B protein | | -2.73 |
| YPO3036 | napC | cytochrome C-type protein NapC | | -3.63 |
| YPO3037 | napB | cytochrome C-type protein NapB precursor | | -4.76 |
| YPO3038 | napA | nitrate reductase catalytic subunit | | -5.59 |
| YPO3039 | napD | assembly protein for periplasmic nitrate reductase | | -4.19 |
| YPO3040 | napF | ferredoxin-type protein NapF | | -3.54 |
| YPO3048 |  | ABC-transporter, ATP-binding protein | | -2.66 |
| YPO3049 |  | binding protein-dependent transport system, inner-membrane component | | -3.89 |
| YPO3050 |  | hypothetical protein | | -3.39 |
| YPO3323 | dmsC | anaerobic dimethyl sulfoxide reductase chain C | | -5.12 |
| YPO3324 | dmsB | anaerobic dimethyl sulfoxide reductase chain B | | -4.02 |
| YPO3325 | dmsA | anaerobic dimethyl sulfoxide reductase chain A | | -2.7 |
| YPO3342 | yhjA | cytochrome C peroxidase | | -2.38 |
| YPO3454 | nrdD | anaerobic ribonucleoside-triphosphate reductase | | -7.32 |
| YPO3455 | nrdG | anaerobic ribonucleoside-triphosphate reductase activating protein | | -7.83 |
| **Sugar transport** | | | |  |
| YPO0410 |  | ABC transporter permease protein | | -2.13 |
| YPO0411 |  | ABC transporter permease protein | | -2.03 |
| YPO0858 |  | sugar transport ATP-binding protein | | -2.11 |
| YPO1508 | mglA | galactose/methyl galaxtoside transporter ATP-binding protein | | -2.44 |
| YPO1509 | mglC | beta-methylgalactoside transporter inner membrane component | | -2.03 |
| YPO1572 |  | sugar transporter | | -2.11 |
| YPO1573 |  | polysaccharide deacetylase | | -2.41 |
| YPO1720 |  | sugar ABC transporter | | -2.49 |
| YPO2255 | araF | L-arabinose-binding periplasmic protein precursor | | -2.06 |
| *YPO2256* | *araG* | *L-arabinose transport ATP-binding protein* | | *-2.18* |
| YPO2257 | araH | L-arabinose transport system permease protein | | -2.06 |
| YPO2581 |  | sugar-binding periplasmic protein | | -2.14 |
| YPO2582 |  | sugar transport ATP-binding protein | | -2.18 |
| YPO2583 |  | sugar transport system permease protein | | -2.05 |
| YPO3016 | nanT | 1. sialic acid transporter | | -2.23 |
| YPO3907 |  | sugar transport system ATP-binding protein | | -2.81 |
| YPO3992 | dctA | C4-dicarboxylate transport protein | | -2.06 |
| YPO4035 |  | sugar transport system permease protein | | -8.11 |
| YPO4036 |  | xylose transporter ATP-binding subunit | | -10.39 |
| YPO4037 | xylF | D-xylose transporter subunit XylF | | -7.37 |
| **Sugar metabolism** | | | |  |
| YPO0407 |  | autoinducer-2 (AI-2) modifying protein LsrG | | -2.67 |
| YPO0408 |  | aldolase | | -3.06 |
| YPO0409 |  | periplasmic solute-binding protein | | -2.79 |
| YPO0852 | bgaB | beta-galactosidase | | -3.85 |
| YPO1291 |  | carbohydrate kinase | | -2.27 |
| YPO1292 | rpiA | ribose 5-phosphate isomerase | | -3.37 |
| YPO1383 | pfl | formate acetyltransferase 1 | | -2.52 |
| YPO1858 |  | N-acetylneuraminic acid mutarotase | | -2.20 |
| YPO2234 | cstA | carbon starvation protein A | | -2.71 |
| YPO2501 | rbsB | sugar binding protein precursor | | -2.08 |
| YPO3020 |  | N-acetylmannosamine kinase | | -2.84 |
| YPO3023 |  | N-acetylmannosamine-6-phosphate 2-epimerase | | -2.58 |
| YPO3024 | nanA | N-acetylneuraminate lyase | | -4.48 |
| YPO3784 |  | carbon starvation protein | | -4.48 |
| YPO4038 | xylA | xylose isomerase | | -5.20 |
| YPO4039 | xylB | xylulose kinase | | -5.50 |
| **Hypothetical** | | | |  |
| YPO0252 |  | hypothetical protein | | -2.25 |
| YPO0485 |  | hypothetical protein | | -2.06 |
| YPO0625 |  | hypothetical protein | | -2.25 |
| YPO1119 |  | hypothetical protein | | -2.51 |
| YPO1387 |  | hypothetical protein | | -2.37 |
| YPO2233 |  | hypothetical protein | | -2.24 |
| YPO3019 |  | hypothetical protein | | -2.09 |
| YPO3480 |  | hypothetical protein | | -2.34 |
| **Other** |  |  | |  |
| YPO0251 | actP | acetate permease | | -2.09 |
| YPO0253 | acs | acetyl-coenzyme A synthetase | | -2.18 |
| YPO0348 | aspA | aspartate ammonia-lyase | | -2.56 |
| YPO0905 | gcvP | glycine dehydrogenase | | -2.46 |
| YPO0906 | gcsH | glycine cleavage system H protein dehydrogenase - | | -2.26 |
| YPO0907 | gcvT | glycine cleavage system aminomethyltransferase T - | | -2.23 |
| YPO1290 | gabD | aldehyde dehydrogenase | | -2.45 |
| YPO1384 | focA | formate transporter | | -2.43 |
| YPO1386 | ansB | L-asparaginase II | | -4.25 |
| YPO1565 | ucpA | Oxidoreductase | | -2.05 |
| YPO1962 | argD | bifunctional succinylornithine transaminase/acetylornithine transaminase | | -2.44 |
| YPO1963 | astA | arginine succinyltransferase | | -2.45 |
| YPO1964 | astD | succinylglutamic semialdehyde dehydrogenase | | -2.23 |
| YPO2269 | bioD | dethiobiotin synthetase ([biotin metabolism](https://en.wikipedia.org/wiki/Biotin_metabolism)) | | -2.18 |
| YPO2502 | gutB | zinc-binding dehydrogenase | | -2.28 |
| YPO2579 |  | pseudo | | -2.50 |
| YPO2662 | dppC3 | binding-protein-dependent transport protein | | -2.32 |
| YPO2967 | dmsC2 | dimethyl sulfoxide reductase chain C protein | | -2.30 |
| YPO2975 | avtA1 | aminotransferase | | -2.24 |
| YPO3322 | torD1 | twin-argninine leader-binding protein DmsD | | -2.43 |
| YPO3034 | maeB | malic enzyme | | -2.00 |
| YPO3047 | ydeN | sulfatase | | -2.35 |
| YPO3479 |  | protease | | -3.29 |
| YPO3925 | argB | acetylglutamate kinase | | -2.20 |
| YPO3927 | argC | N-acetyl-gamma-glutamyl-phosphate reductase | | -2.04 |
| YPO4033 |  | phage integrase | | -2.05 |
| YPO4034 |  | AraC-family transcriptional regulatory protein | | -8.09 |

^1^Gene name and annotation is according to *Y. pestis* CO92 strain (NC_003143.1).

The annotation was inferred from *Y. pestis* CO92 genome sequencing project and was complemented by annotations from orthologous sequences of *Y. pestis* KIM strain (NC_004088.1) and *E. coli* K12 (NC_000913) (Expectation value < e^-10^ and alignment over 80 % of the protein sequence).

Additional annotation data was based on the KEGG website (<http://www.genome.jp/kegg/>) and the Transporter Proteins database of *Yersinia pestis* CO92 (<http://www.membranetransport.org/transporter2.php?oOID=ypes1>

^2^FC-fold changes.
