# Supplementary material for: Disruption of the NlpD lipoprotein of the plague pathogen Yersinia pestis affects iron acquisition and the activity of the twin-arginine translocation system
Source: PLoS Negl Trop Dis. 2019 Jun 6;13(6):e0007449. doi: 10.1371/journal.pntd.0007449 (PMC6553720; doi:10.1371/journal.pntd.0007449)
Supplement: S6 Table — (DOCX) [file pntd.0007449.s013.docx]

Table S6 qRT-PCR-determined differentially expressed genes between wild-type and *nlpD* mutant.

| PID^1^ | Gene Name | FC ^2^ | | Log of FC ^2^ | |
| --- | --- | --- | --- | --- | --- |
|  |  | qRT-PCR | array | qRT-PCR | array |
| YPO0075 | *cpxP* | 11.4 | 15.8 | 1.056905 | 1.198657 |
| YPO0723 | *flgC* | 6.3 | 2.2 | 0.799341 | 0.342423 |
| YPO0956 | YPO0956 | 2.8 | 4.6 | 0.447158 | 0.662758 |
| YPO1045 | *tsf* | 1 | 1 | 0 | 0 |
| YPO1301 | *psaE* | 3.7 | 3 | 0.568202 | 0.477121 |
| YPO1302 | *psaF* | 6.4 | 2 | 0.80618 | 0.30103 |
| YPO1415 | *pyrD* | 1 | 1 | 0 | 0 |
| YPO1715 | *ybjR* | 3.5 | 3.2 | 0.544068 | 0.50515 |
| YPO1907 | *irp5* | 1.7 | 2.5 | 0.230449 | 0.39794 |
| YPO2739 | *ccmF* | 0.3571 | 0.22727 | -0.44721 | -0.64346 |
| YPO3355 | *appR* | 1 | 1 | 0 | 0 |
| YPO3356 | *nlpD* | 0.555 | 0.3846 | -0.25571 | -0.41499 |
| YPO4034 | YPO4034 | 0.22727 | 0.12345 | -0.64346 | -0.90851 |

^1^ PID - YPO gene number according to *Y. pestis* CO92 strain.

^2^ FC-fold change.
